# Direct control of electron spin at an intrinsically chiral surface for highly efficient oxygen reduction reaction

**DOI:** 10.1073/pnas.2413609122

**Published:** 2025-02-25

**Authors:** Xia Wang, Mayra Peralta, Xiaodong Li, Paul V. Möllers, Dong Zhou, Patrick Merz, Ulrich Burkhardt, Horst Borrmann, Iñigo Robredo, Chandra Shekhar, Helmut Zacharias, Xinliang Feng, Claudia Felser

**Affiliations:** ^a^Department of Topological Quantum Chemistry, Max-Planck-Institute for Chemical Physics of Solids, Dresden 01187, Germany; ^b^Center for Advancing Electronics Dresden, Technische Universität Dresden, Dresden 01062, Germany; ^c^Faculty of Chemistry and Food Chemistry, Technische Universität Dresden, Dresden 01062, Germany; ^d^Center for Soft Nanoscience, University of Münster, Münster 48149, Germany; ^e^Tsinghua Shenzhen International Graduate School, Tsinghua University, Shenzhen 518055, China; ^f^Donostia International Physics Center, Donostia-San Sebastian 20018, Spain; ^g^Department of Synthetic Materials and Functional Devices, Max Planck Institute of Microstructure Physics, Halle 06120, Germany

**Keywords:** spin polarization, oxygen reduction reaction, intrinsic homochiral catalysts, topology, spin–orbital coupling

## Abstract

The oxygen reduction reaction (ORR) in acidic media suffers from sluggish kinetics, primarily due to the spin-dependent electron transfer involved. The direct generation of spin-polarized electrons at catalytic surfaces remains elusive, and the underlying mechanisms are still controversial due to the lack of intrinsically chiral catalysts. To address this challenge, we investigate topological homochiral PdGa (TH PdGa) crystals with intrinsically chiral catalytic surfaces for ORR. Through spin-resolved photoemission spectroscopy and theoretical simulations, we show that both structural chirality and spin–orbit coupling are critical for inducing spin polarization at the surface of TH PdGa. As a result, TH PdGa achieves a kinetic current density over 100 times higher than the achiral PdGa (AC PdGa) at 0.85 V versus the reversible hydrogen electrode. This work underscores the pivotal role of spin polarization in enhancing acidic ORR activity and lays the groundwork for the rational design of chiral catalysts for spin-dependent catalysis.

## Significance

The spin-dependent electron transfer process results in sluggish kinetics for oxygen reduction reaction (ORR) in acidic media, limiting the efficiency of energy conversion systems like metal–air batteries and fuel cells. Controlling electron spin offers an alternative approach to enhance ORR activity, potentially surpassing the limitations of the volcano plot. However, directly generating spin polarization at catalytic surfaces remains elusive, with the underlying mechanism under debate due to the lack of intrinsically homochiral catalysts. This study uses topological homochiral PdGa crystal to improve ORR kinetics and selectivity by controlling electron spin at its instinct chiral surface. We investigate the role of chirality-driven quantum properties, revealing that both structural chirality and spin–orbit coupling are key to generating spin polarization in chiral materials.

The electrocatalytic oxygen reduction reaction (ORR) plays a critical role in determining the efficiency of sustainable energy conversion systems, such as metal–air batteries and fuel cells (1, 2). However, the sluggish kinetics of the ORR, especially in acidic media, significantly limits the performance of these devices (3–5). The ORR involves a four-electron transfer process, starting from oxygen molecules in triplet states (^3^Σ–g), while oxygen species and reaction products are generated in singlet ground states (1A). Due to the nonconservation of electron spin during the conversion of triplet oxygen molecules, additional energy is required, as this step is forbidden by quantum mechanics (6). Therefore, controlling and understanding the spin-dependent electron transfer process is essential for improving ORR efficiency, yet this aspect has remained largely neglected.

The chiral-induced spin selectivity (CISS) effect opens a way to manipulate the electron spin in oxygen catalysis (7–10). In particular, CISS affects the spin of electrons transferred through chiral structures, thereby influencing reaction kinetics and pathways (11, 12). As a result, CISS provides a promising approach to enhance ORR activity, potentially surpassing the limitations imposed by the volcano plot (6, 13, 14). However, previously chiral oxygen catalysts are obtained by imparting chirality to initially achiral metal surfaces through the adsorption of chiral molecules or the formation of imprinted chiral cavities (12, 15, 16). While these strategies introduce chirality, the insulating nature of the chiral molecules on the catalytic surfaces reduces the accessibility of active sites and lowers the overall conductivity of the electrode. Moreover, the mechanism by which polarized electrons are transferred from the chiral molecules to the catalytic active sites remains unclear (17). In addition, these “artificial chiral surfaces” complicate the investigation of the underlying mechanisms of spin polarization in chiral catalysts for ORR. Therefore, the development of intrinsically chiral electrocatalysts with well-defined homochiral structures is paramount to understand the spin-dependent electron transfer process in ORR and thus to reveal the interplay between catalytic activity and chirality-driven quantum properties such as spin–orbital coupling (SOC) and exotic spin/orbital angular momentum (18, 19).

The topological homochiral PdGa (TH PdGa) crystal, with space group *P*2_1_3 (No. 198), is a unique material featuring intrinsically chiral geometric and electronic structures (20, 21). The electron spin at the crystal surface exhibits a spin–orbital momentum locking state, owing to the substantial SOC (22). The chiral electronic structure of TH PdGa is characterized by chiral fermions and Fermi-arc with reversed velocities (23). Moreover, TH PdGa exhibits robust topological surface states (TSS) that are resistant to extrinsic modifications, such as doping, strain, and defects, making it an ideal platform to study spin-dependent electron transfer processes in the ORR (24–26). In this work, we demonstrate the critical role of spin polarization in enhancing ORR kinetics and efficiency using single crystal of TH PdGa as a proof-of-concept electrocatalyst. The generation of spin polarization in TH PdGa is experimentally probed by spin-resolved photoemission measurements, which reveal opposite spin polarization values for the two TH PdGa enantiomers (denoted TH PdGa-A and TH PdGa-B). Theoretical modeling further underscores the importance of both the chiral structure and the SOC in generating of spin polarization in TH PdGa. As a result, TH PdGa exhibits exceptional ORR performance, including a record-high half-wave potential (*E_1/2_*) of 0.90 V versus reversible hydrogen electrode (RHE), a large kinetic current density of 156 mA cm^−2^ at 0.85 V versus RHE, and a turnover frequency (TOF) of 18.58 s^−1^. This performance significantly surpasses that of achiral PdGa (AC PdGa), which exhibits an *E_1/2_* of 0.79 V versus RHE, a kinetic current density of 1.53 mA cm^−2^ at 0.85 V versus RHE, and a TOF of 0.23 s^−1^, respectively. In addition, TH PdGa demonstrates the highest ORR selectivity compared to the (111) surface of Pd single crystal (denoted SC Pd), the (111) surface of Pt single crystal (denoted SC Pt), and AC PdGa. This work represents a breakthrough in the development of high-performance intrinsic chiral catalysts for acidic ORR by directly controlling the electron spin at the catalytic surface, enabling further progress in next-generation energy conversion technologies.

## Results

### Structure Characterization of TH PdGa.

**Fig. 1.**
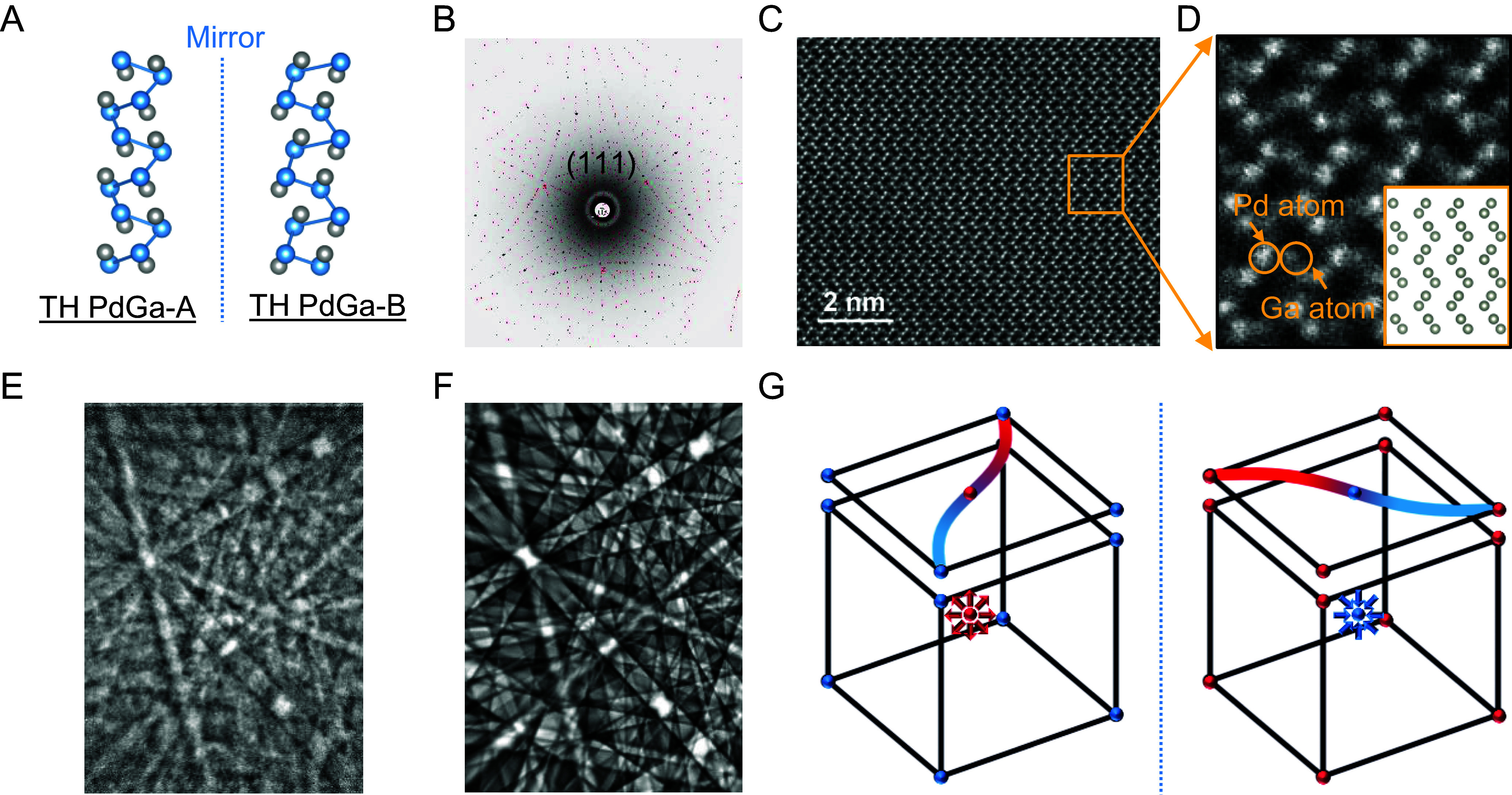
(*A*) Illustration of the chiral crystal structure of both TH PdGa-A and TH PdGa-B enantiomers. Blue and gray dots represent Pd and Ga atoms, respectively. (*B*) Laue X-ray diffraction pattern (black dots) of the TH PdGa-A crystal, showing a single simulated (111) pattern (red dots). (*C*) STEM image of TH PdGa-A. (*D*) Zoomed-in image of the yellow rectangle in panel *C*. The *Inset* shows the arrangement of Pd atoms along the [101] direction. White dots correspond to Pd atoms, and gray dots next to Pd atoms represent Ga atoms. (*E*) Electron backscatter diffraction (EBSD) pattern of the TH PdGa-A at the EBSD01 site. (*F*) Simulated EBSD pattern of the TH PdGa-A at the EBSD01 site. (*G*) Schematic of the chiral electronic structure of both TH PdGa enantiomers, including chiral fermions (red and blue) in the bulk BZ and Fermi-arcs in the surface BZ. The red and blue spheres represent Weyl fermions with opposite chiral charges.

The TH PdGa crystal in this study belongs to the chiral B20 cubic space group (No.198). Its chiral structure is distinguished by the helical arrangement of Pd or Ga atoms along the [111] direction, with TH PdGa-A and TH PdGa-B enantiomers exhibiting either a clockwise or anticlockwise helix (Fig. 1*A*). In addition, the topmost surface layers of Pd or Ga trimers on the threefold symmetric (111) crystal plane exhibit opposite handedness, enabling the surface chirality, and influencing the surface properties of TH PdGa (*SI Appendix*, Fig. S1).

Bulk single crystals of TH PdGa, up to several centimeters in size, were grown by the self-flux method (*Materials and Methods*). The chemical composition of the as-grown TH PdGa crystals was confirmed by elemental mapping, showing a Pd/Ga ratio of 1 (*SI Appendix*, Fig. S2*A*). Single crystal X-ray diffraction (XRD) and the Flack method were used to analyze the crystal quality and structure. The single-crystal XRD patterns of TH PdGa-A obtained along the c-axis (*SI Appendix*, Fig. S2*B*) showed sharp, symmetrical diffraction spots, indicating excellent crystal quality, with a lattice constant of 4.8967 Å. Further details on the single-crystal XRD analysis of TH PdGa can be found in our previous work (23, 27). Two pieces of plate-like TH PdGa-A and TH PdGa-B crystals were prepared by cutting the as-grown TH PdGa crystal rods along the [111] direction. This was confirmed by the XRD patterns showing prominent peaks corresponding to the (111) and (222) planes (*SI Appendix*, Fig. S2*C*). Laue X-ray diffraction was performed on both TH PdGa-A and TH PdGa-B enantiomers, revealing a single pattern with threefold rotation symmetry along the [111] direction (Fig. 1*B* and *SI Appendix*, Fig. S2*D*). This indicated the presence of single crystallinity and homochirality without any twinning or domains in TH PdGa-A and TH PdGa-B. To gain a deeper insight into the atomic arrangement of TH PdGa, a thin lamella sample was prepared with TH PdGa-A for scanning transmission electron microscopy (STEM) using focused ion beam technique. In the STEM images, white dots represent heavy Pd atoms, and gray dots adjacent to Pd atoms correspond to Ga atoms (Fig. 1 *C* and *D*). The Pd–Ga atomic stacking sequence matched well with the simulated atomic arrangement along the [101] direction, with 100% occupancy of Pd and Ga sites (Fig. 1 *D*, *Inset*). The handedness of TH PdGa was distinguished by EBSD, where each TH PdGa crystal (TH PdGa-A and TH PdGa-B) showed only one chirality at different detection spots, further confirming the homochirality of the entire crystal surface (Fig. 1 *E* and *F* and *SI Appendix*, Figs. S3 and S4). Furthermore, Fig. 1*G* shows a schematic of two oppositely charged (chiral) Weyl fermions in the bulk and a reversal of the propagation direction of the Fermi-arc at the surface Brillouin zone (BZ). The multifold fermions at the Γ and R points act as sources (positive Chern number) or sinks (negative Chern number) of the Berry curvature, indicating the chiral electronic structure of TH PdGa (23).

### ORR Reactivity and Selectivity of TH PdGa.

**Fig. 2.**
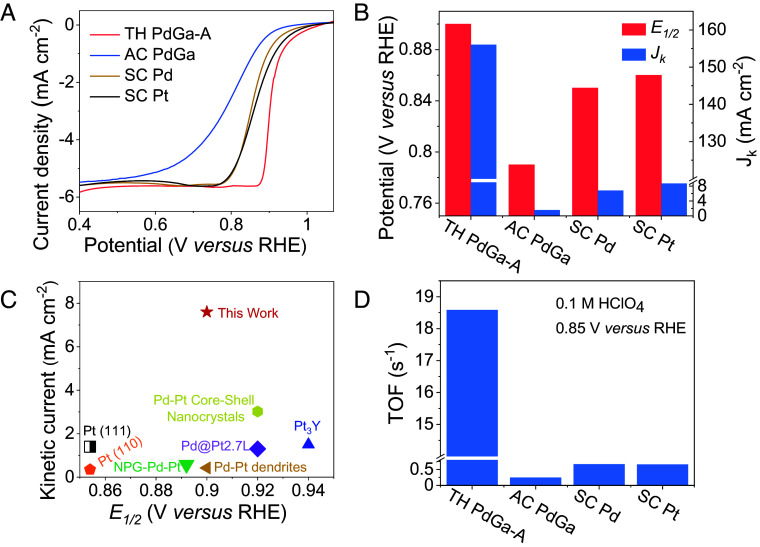
(*A*) LSV polarization curves of TH PdGa-A, AC PdGa, SC Pd, and SC Pt. (*B*) *E_1/2_*and *J_k_* at 0.85 V versus RHE for TH PdGa-A, AC PdGa, SC Pd, and SC Pt. (*C*) Comparison of *E_1/2_*and *J_k_* of TH PdGa-A with reported state-of-the-art noble metal-based ORR catalysts in acidic media (28–33). Note: The *J_k_*values in panel *C* are calculated at 0.9 V versus RHE. (*D*) Comparison of TOF values of TH PdGa-A, AC PdGa, SC Pd, and SC Pt for ORR at 0.85 V versus RHE.

The ORR performance of TH PdGa was evaluated in 0.1 M aqueous HClO_4_ solution by employing bulk TH PdGa crystal as the electrode directly (*SI Appendix*, Fig. S5). SC Pd, SC Pt, and AC PdGa were used as reference samples for comparison. AC PdGa exhibits a polycrystalline structure (*SI Appendix*, Figs. S6 and S7). Linear sweep voltammetry (LSV) was performed to evaluate the ORR activity. As shown in Fig. 2*A*, TH PdGa-A demonstrates superior ORR activity, exhibiting the highest onset potential (*E_onset_*) and *E_1/2_*. In the mixed kinetic-diffusion control region between 0.8 and 1.0 V, ORR kinetics on TH PdGa-A are accelerated compared to SC Pd, resulting in a 50 mV positive shift in *E_1/2_*. TH PdGa-B showed similar ORR activity to TH PdGa-A (*SI Appendix*, Fig. S8), and therefore, TH PdGa-A was chosen for further testing and analysis in this work. The kinetic current density (*J_k_*) at 0.85 V versus RHE was calculated, with TH PdGa-A exhibiting the highest *J_k_*of 156 mA cm^−2^ (Fig. 2*B*). This value is over 20 times higher than that of SC Pd, despite SC Pd having more active sites. When compared to the benchmark Pt/C with a larger surface area, TH PdGa-A also outperforms in terms of *E_onset_, E_1/2_,* and *J_k_*(*SI Appendix*, Fig. S9). The Tafel slope, a key parameter for assessing ORR kinetics, reveals that TH PdGa-A has the smallest Tafel slope (84.2 mV dec^−1^) compared to the other catalysts (*SI Appendix*, Fig. S10), further suggesting that the TH PdGa-A exhibits the fastest ORR kinetics. A comprehensive comparison of ORR activity in acidic conditions is presented in Fig. 2*C*, highlighting the superior activity of TH PdGa-A compared to previously reported noble metal-based ORR catalysts. To better understand the intrinsic reactivity, the ORR activities were further analyzed through deconvolution into the intrinsic TOF. TH PdGa-A demonstrated an impressive TOF of 18.58 s^−1^, exceeding the TOF of SC Pd and AC PdGa by more than 28 times and 80 times, respectively (Fig. 2*D*). These results emphasize the superior activity of TH PdGa-A over achiral catalysts. The stability of TH-PdGa-A was tested using accelerated durability test-cyclic voltammetry and chronoamperometry measurements, indicating excellent stability for ORR in acidic condition (*SI Appendix*, Fig. S11). Additionally, the crystallinity, composition, and electronic states of Pd on the TH-PdGa-A surface were fully preserved after the ORR test (*SI Appendix*, Figs. S12 and S13), further confirming its exceptional structure stability for ORR.

**Fig. 3.**
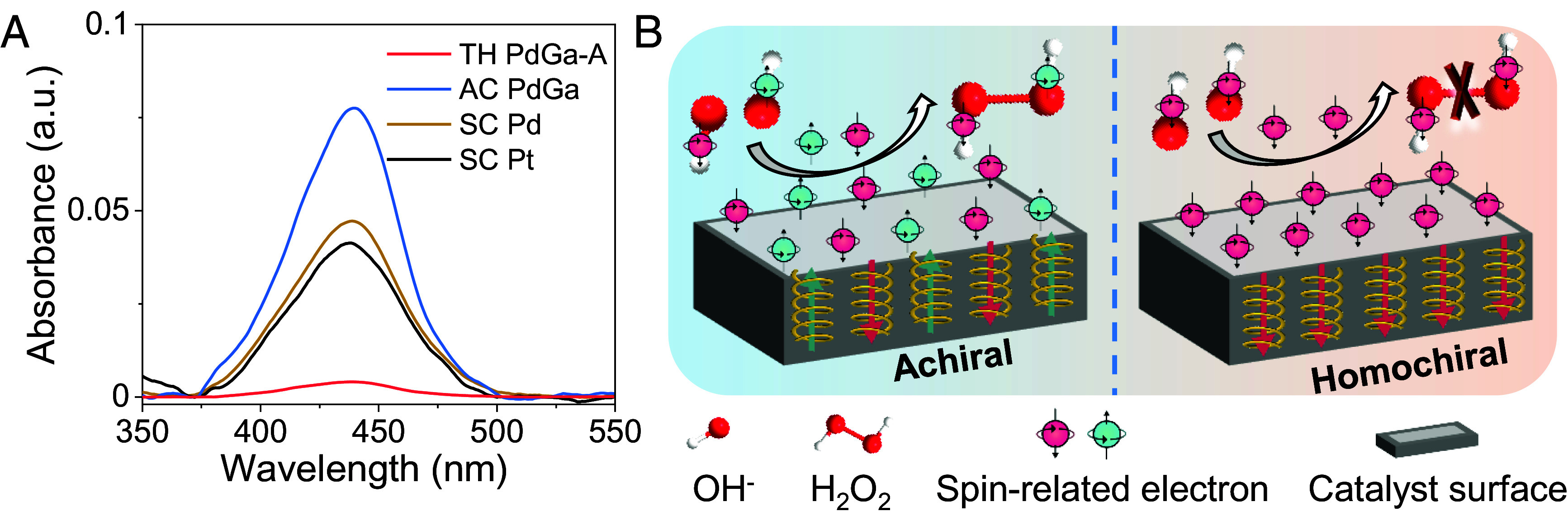
(*A*) UV-visible spectrophotometric analysis of H_2_O_2_ production from chronoamperometry at 0.5 V versus RHE on TH PdGa-A, AC PdGa, SC Pd, and SC Pt. (*B*) Illustration of the spin polarization induced by the homochiral crystal structure for enhanced ORR selectivity. In an achiral catalyst, the unpaired electrons on the two OH• intermediates are aligned antiparallel, allowing interaction on a singlet surface and facilitating the formation of H_2_O_2_. In contrast, on a homochiral catalyst, the spins of the two electrons are aligned parallel, resulting in interaction on a triplet surface, which inhibits the formation of H_2_O_2_ and favors the four-electron ORR process.

The selectivity of the ORR was evaluated by monitoring the production of H2O2, a key indicator of the reaction pathway, as its formation is expected to be reduced on chiral catalyst due to the CISS effect (8, 11). To measure the H_2_O_2_ yield, UV-visible spectrophotometry was employed after a chronoamperometric test. The results showed a significant reduction in the H_2_O_2_ yield for TH PdGa-A, which can be attributed to the fact that the formation of H_2_O_2_ (a singlet species) is spin-forbidden on a spin-polarized chiral surface (Fig. 3*A*). This phenomenon is consistent with the previously observed CISS effect in water splitting (12, 15, 34, 35). During the ORR process, the OH intermediates formed from O_2_ will exhibit different spin states depending on the chirality of the catalyst surface. On an achiral catalyst surface, the unpaired electrons of OH display mixed spin states. As a result, the OH intermediates in their doublet ground state can interact with each other, allowing for the formation of H_2_O_2_. On a homochiral catalyst surface, however, the unpaired electrons of the OH intermediates align in a parallel fashion, making the formation of H_2_O_2_ symmetry forbidden (Fig. 3*B*). This intriguing behavior highlights the role of the spin polarization in governing the selectivity of the ORR and sheds light on the unique catalytic properties of the TH PdGa. Importantly, the induced spin polarization aligns either parallel or antiparallel to the direction of the applied electric current, independent of the crystalline or molecular axis. As a result, when electrons travel through the bulk chiral crystal and reach the electrode surface, the spin polarization is always oriented along the direction of the current. Consequently, the transition states at the electrode–electrolyte interface experience the same spin polarization, even if the chiral crystal involves multiple facets with various orientation.

### Electronic Structure and Spin Polarization in TH PdGa.

**Fig. 4.**
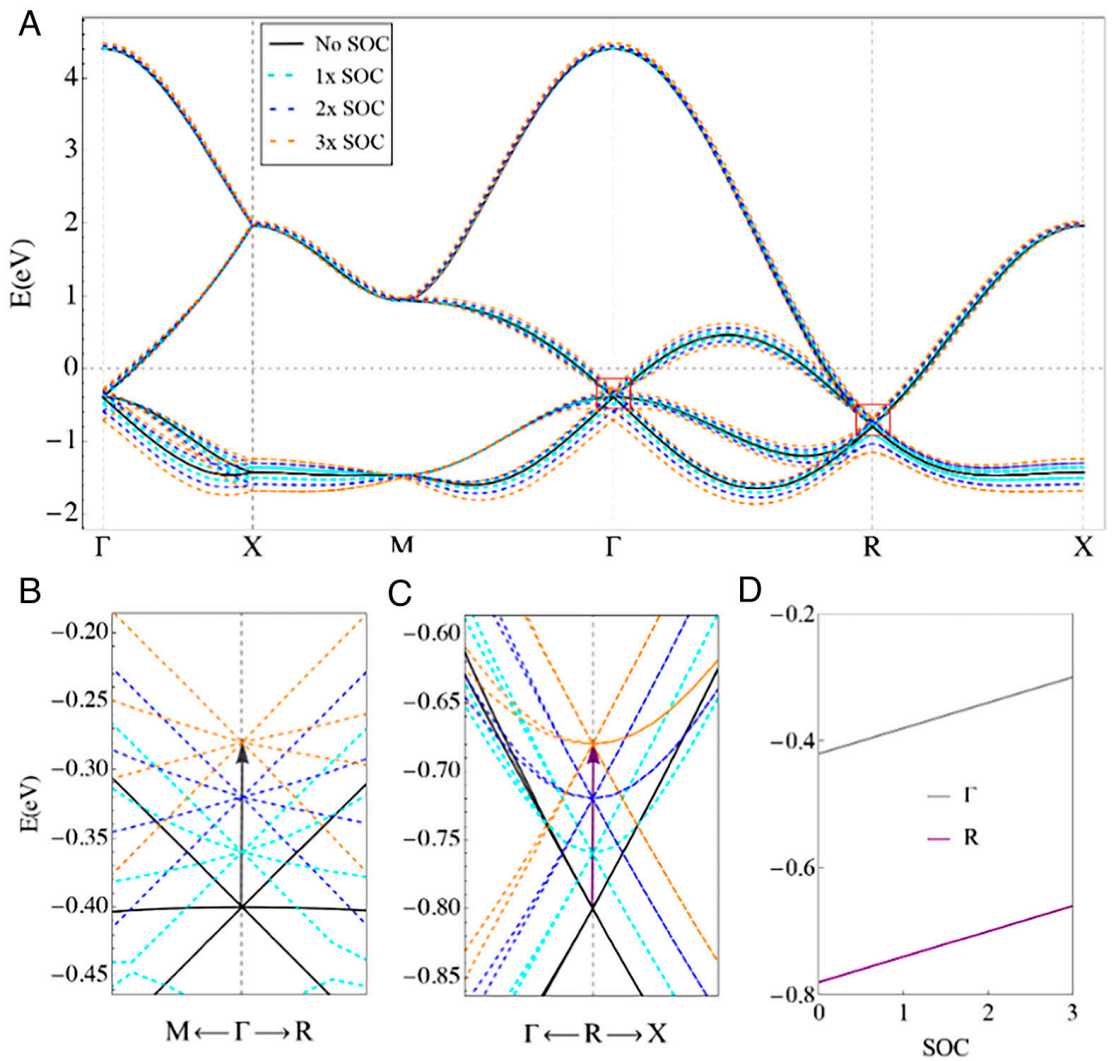
(*A*) Band structure of TH PdGa with varying strengths of SOC (No SOC, 1 × SOC, 2 × SOC, and 3 × SOC). Zoomed-in figures around the multifold fermions at (*B*) the G point, and (*C*) the R point. As the SOC strength increases, both Weyl points are approaching the Fermi level. (*D*) Positions of the multifold fermions at the G and R points relative to the Fermi level as a function of SOC.

The electronic structure plays a crucial role in catalytic performance. Fig. 4*A* presents the bulk band structure of TH PdGa with varying SOC strengths throughout the BZ. The presence of SOC and the absence of inversion symmetry lead to observable band splitting with opposite spin at the G and R high symmetry points in the bulk. In particular, a fourfold degenerate Rarita–Schwinger–Weyl spin 3/2 fermion with a Chern number +4 is found at the G point, while two threefold degenerate double-Weyl spin 1 fermions with Chern numbers of –4 are located at the R points (*SI Appendix*, Fig. S14) (23). The substantial SOC in TH PdGa causes the G and R Weyl points to approach the Fermi level, which is predicted to benefit catalytic reactions by enriching electrons and weakening the bonding strength of oxygen intermediates (Fig. 4 *B*–*D*) (36). Moreover, the majority of carriers at the Fermi surface are mainly contributed by Pd, suggesting that the Fermi-arc TSS are primarily derived from d orbitals of Pd (*SI Appendix*, Fig. S15), which are believed to increase the reservoir of catalytically active electrons in the ORR process.

**Fig. 5.**
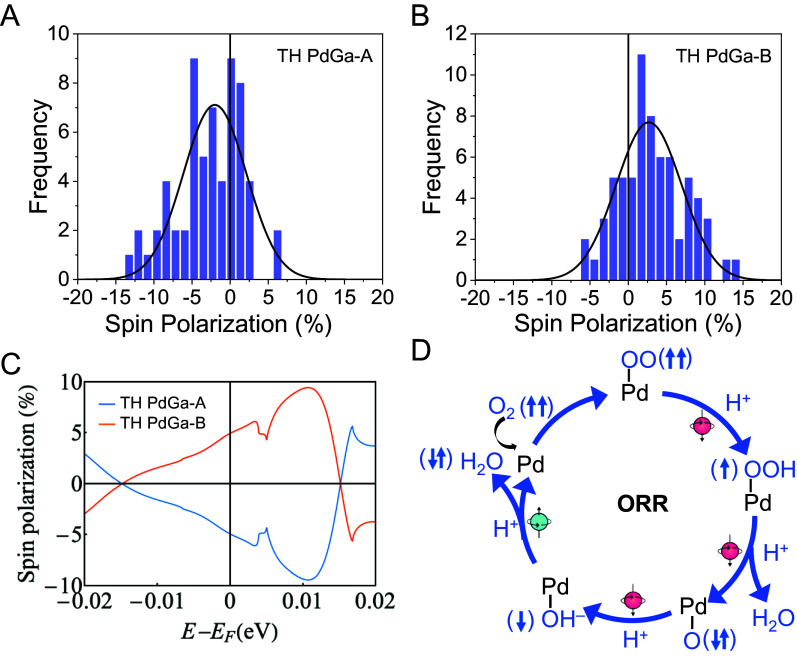
Photoelectron spin polarization distributions measured on (*A*) TH PdGa-A and (*B*) TH PdGa-B surfaces. Photoelectrons were excited with linearly polarized light. The average spin polarization values were determined by fitting Gaussian functions to the distributions. (*C*) Spin polarization calculated using Eq. **1**, with an inset around the Fermi level, where the polarization is −4.92% for TH PdGa-A and +4.92% for TH PdGa-B respectively. (*D*) Illustration of the spin-dependent electron transfer process during ORR on the TH PdGa surface.

Spin polarizations in TH PdGa were directly probed using spin-resolved photoemission measurements (*SI Appendix*, Fig. S16) (37–39). Fig. 5 *A* and *B* show spin polarization distributions on the (001) surfaces of two TH PdGa enantiomers with opposite chirality. Each count in the histograms corresponds to approximately 10^4^ detected electrons. The average spin polarizations were determined to be *P* = (−2.0 ± 4.1)% for TH PdGa-A and *P* = (+2.7 ± 4.3)% for TH PdGa-B. Given that the charge carrier density in TH PdGa is on the order of 10^22^ cm^−3^ (*SI Appendix*, Fig. S17), the spin polarization concentration in TH PdGa is approximately 10^7^ times greater than that in chiral molecule–modified catalytic surfaces (40–43). In materials with strong SOC, spin-polarized photoelectrons can also be excited by circularly polarized light. These nonzero spin polarizations arise due to the selection rules that govern specific final spin states within a nondegenerate band structure. It is important to note that the spin polarization values shown in Fig. 5 *A* and *B* were measured using linearly polarized light, with light incidence and electron emission along the surface. This means that the measured spin polarization values directly reflect the intrinsic, chirality-induced symmetry breaking of the TH PdGa surfaces. In contrast, the spin polarization distributions on AC PdGa, determined using a Gaussian fit, yielded *P* = (–0.2 ± 3.3)% (*SI Appendix*, Fig. S18). This is likely due to the polycrystalline structure of AC PdGa, which contains both enantiomers, resulting in a mixture of spin up and spin down electrons. This interpretation is further supported by the correlation between the sign of the spin polarization and the specific PdGa enantiomer, as expected from symmetry considerations (19). In addition to the nonzero photoelectron spin polarization, chiral materials can exhibit a circular dichroism in the photoelectron yield. To evaluate this, the light polarization was alternated between s-linear, clockwise (cw), and counterclockwise (ccw) circular light during the measurement. For TH PdGa-A and TH PdGa-B, circular dichroism values of ΔI ≈ (−22.6 ± 2.1)% and ΔI ≈ (+9.6 ± 0.3)% were measured, respectively. This circular dichroism asymmetry further highlights the pronounced chiral characteristics and intrinsic chirality-induced effects in the TH PdGa crystals. In contrast, the circular dichroism for AC PdGa is ΔI ≈ (1.95 ± 2.77)%, indicating no significant deviation from zero within the margin of error. These results further emphasize the crucial role of homochiral structure in generating spin polarization in the crystal.

Theoretical modeling was applied to calculate spin-dependent transmission in TH PdGa (*SI Appendix*, Note S1) (44–47). The spin polarizations for TH PdGa-A and TH PdGa-B around the Fermi level were −4.92% and +4.92%, respectively (Fig. 5C and *SI Appendix*, Fig. S19). The intergradation of SOC is found to be necessary for generating the observed spin asymmetry during the theoretical calculation. To further assess the impact of the chiral structure, additional calculations revealed a pronounced contrast in spin asymmetries between the chiral (4.25%) and achiral (0.1%) structures (*SI Appendix*, Note S1 and Fig. S20). These results underscore the crucial role of both SOC and the chiral structure in inducing and enhancing spin asymmetry in TH PdGa. The mechanism of spin polarization in TH PdGa for enhanced ORR kinetics is illustrated in Fig. 5*D*. During the ORR process, oxygen molecules in their triplet ground state must split to form water in the singlet state. On the homochiral surface, the electrons on the triplet potential surface can more easily transfer through the conduction band of TH PdGa to the O_2_‚ compared to the achiral surface, which lacks spin control. Importantly, the spin polarization is always along the current direction, regardless of the crystalline axis or the direction of the adsorbed molecules. As a result, the ORR kinetics and efficiency are significantly improved on the TH PdGa crystal surface.

## Discussion

In conclusion, this study introduces TH PdGa as a homochiral catalyst for the ORR in acidic media, which exhibits significantly enhanced reaction kinetics due to spin polarization at the intrinsic chiral surfaces. The TH PdGa catalysts demonstrate remarkable performance, with *E_1/2_* of 0.90 V, a kinetic current density of 156 mA cm^–2^ at 0.85 V versus RHE, and an intrinsic catalytic TOF of 18.58 s^–1^. These results outperform AC PdGa by more than 100 times in terms of kinetic current density, while also rivaling other state-of-the-art catalysts in acidic electrolytes. The spin polarization in TH PdGa crystals is directly probed by spin-resolved photoemission experiments and theoretical modeling, which show opposite spin polarization values on the surface of TH PdGa-A and TH PdGa-B. Importantly, it is confirmed that both structure chirality and SOC play crucial roles for generating spin-polarized electrons in TH PdGa. This work provides valuable insights into the quantum mechanisms of spin polarization in bulk chiral solids. The experimental and theoretical findings underscore the potential of topological chiral materials as next-generation spin-dependent catalysts, offering high efficiency and advanced selectivity for catalytic applications.

## Materials and Methods

### Materials Preparation.

The TH PdGa single crystals were grown using the self-flux technique (23). First, a polycrystalline ingot was prepared through arc melting stoichiometric amounts of Pd and Ga metals (99.99% purity) in an argon atmosphere. Second, the as-prepared ingot was crushed into powder, filled in an alumina crucible, and sealed in a quartz tube. Third, the quartz ampoule was heated to 1,100 °C at a rate of 100 °C /h and annealed at 1,100 °C for 12 h. The temperature was then slowly reduced to 900 °C at a rate of 1.5 °C/h, followed by further cooling to 800 °C at a rate of 20 °C/h, where it was held for 72 h before cooling to room temperature at a rate of 20 °C/h. The obtained TH PdGa single crystals were cut into a plate-like shape with a smooth geometry surface area, which was used directly as the electrode for catalysis. The surface area of the TH PdGa electrode was accurately measured using a high-magnification stereomicroscope (Olympus SZX16). The measurement process was repeated three to five times to ensure consistency and reliability. To prepare the AC PdGa, the same ingot used for TH PdGa was crushed to powder, and particles larger than 20 microns were removed by sieving. The fine powder was pressed into a pellet, wrapped in tantalum foil and sealed in a quartz ampoule under vacuum. The AC PdGa was obtained by annealing at 700 °C for 72 h. SC Pd (111) and SC Pt (111) were purchased from MaTeck Material Technologie & Kristalle GmbH. Pt/C was purchased from Sigma-Aldrich.

### Materials Characterization.

A white beam backscattering Laue X-ray diffraction was first performed to check the single crystallinity of TH PdGa at room temperature. Single-crystal XRD data were collected on a Rigaku AFC7 four-circle diffractometer with a Saturn 724+ CCD-detector, employing graphite-monochromatized Mo-Kα radiation. A FEI Tecnai G2 F30 electron microscope with an acceleration voltage of 300 kV was used to perform STEM. UV-Vis absorption spectra were recorded on an Agilent Cary 5000 UV-Vis-NIR spectrophotometer with an integration sphere. Powder X-ray diffraction was carried out on a STOE STADI P diffractometer with Cu Kα target (λ = 1.54178 Å).

### Electrochemical Measurements.

Electrochemical measurements were carried out on a PINE electrochemical workstation using a standard three-electrode configuration. A Pt electrode and an Ag/AgCl electrode were used as the counter and reference electrode, respectively. For measurements of TH PdGa, AC PdGa, SC Pd, and SC Pt, a piece of bulk crystal was mounted on the commercial rotator (PINE Instruments) and used as the working electrode. For the Pt/C, 1 mg Pt/C (20% Pt/C, Sigma-Aldrich) was dispersed in 1.0 mL of solution containing 480 μL of ethanol, 480 μL of H_2_O, and 40 μL of 5% Nafion solution. The mixture was ultrasonicated for 30 min to form homogeneous catalyst ink. Then, 15 μL of the Pt/C catalyst ink was drop-cast on the glassy carbon rotating disk electrode (RDE, 5 mm in diameter) for electrocatalysis. The ORR performance was assessed by recording CV scans from 1.1 V to 0.3 V at 10 mV s^−1^ in O_2_-saturated electrolytes with the positive-going curves being used for activity determination and iR correction. The scan and rotation rates for ORR measurements were 10 mV s^−1^ and 1,600 rpm, respectively. The accelerated durability test was performed by potential cycling from 0.60 to 1.00 V versus RHE at a sweep rate of 100 mV s^−1^.

The electrochemical surface area (ECSA) was calculated from CV measurements in the non-Faradaic region at scan rates from 10 to 60 mV s^−1^. The ECSA was determined using the formula:

[1] $$\text{ECSA}=\frac{C_{dl}}{C_{s}},$$

where *C_dl_* is the double layer capacitance obtained from the average slopes of the charging and discharging current density versus scan rate plot, and *Cs* is the specific capacitance of the material. A specific capacitance value of 40 µF cm^−2^ was used in this study.

The specific activity was calculated by normalizing the kinetic current to the corresponding geometric surface area, assuming a roughness factor of 1 for all single-crystal electrodes.

To measure the H_2_O_2_ yield, a chronoamperometric test was employed by controlling the total reduction charge to 1 mA h.

The Nernst equation was used to convert potentials from the Ag/AgCl scale to the RHE scale:

[2] $$E_{\text{RHE}}=E_{\text{Ag}/\text{AgCl}}+0.059\times \text{pH}+0.197,$$

The kinetic current density (*J_K_*) was obtained from LSV measurements based on the Koutecky–Levich equation:

[3] $$\frac{1}{J}=\frac{1}{J_{L}}+\frac{1}{J_{K}},$$

where the diffusion limiting current density *J_L_* was chosen at potential 0.4 V versus RHE.

### Turnover Frequency Calculations.

The (111) crystal plane of TH PdGa are regarded as the active surface, with an active site density (*D*) of 7.02 × 10^14^ atoms cm^−2^. The TOF was calculated using the kinetic current activity *J_K_* (mA cm^−2^) at a given electrode potential (0.85 versus RHE).

The total number of oxygen atom turnovers *TO* was calculated from the current density as follows:

[4] $$TO=\left(j\frac{\mathrm{mA}}{{\mathrm{cm}}^{2}}\right)\left(\frac{1\;Cs^{-1}}{1,000\;mA}\right)\left(\frac{1\;mol\;e^{-1}}{96,485.3C}\right)\left(\frac{1\;mol\;O_{2}}{4\;mol\;e^{-1}}\right)\left(\frac{6.023\times 10^{23}\;O_{2}}{1\;mol\;O_{2}}\right)=1.56\times 10^{15}\frac{O_{2/}s}{{\mathrm{cm}}^{2}}per\frac{\mathrm{mA}}{{\mathrm{cm}}^{2}},$$

The TOF is then calculated using the following equation:

[5] $$TOF\;\left[\mathrm{electron}\;{\mathrm{site}}^{-1}\;s^{-1}\right]=\frac{T_{O}\times J_{K}}{D\times A_{\mathrm{ECSA}}},$$

where *A*_ECSA_ is the ESCA.

### Photoelectron Spin Polarization Measurements.

A simplified scheme of the Mott scattering setup is provided in *SI Appendix*, Fig. S16. Deep-UV laser pulses at λ = 213 nm (*hν* = 5.83 eV) with a pulse length of about 200 ps were used. The radiation is generated as the fifth harmonic of a Nd: YVO_4_ laser through frequency doubling in a lithium triborate (LBO) crystal, followed by two consecutive frequency mixing steps in a second LBO crystal and a β-barium borate (BBO) crystal. The laser pulses impinge onto the sample surface along the surface normal, with a repetition rate of 20 kHz. Photoelectrons are collected along the surface normal using electron-optical elements and guided into the Mott polarimeter. The polarimeter consists of a 70 nm thin gold foil set to a potential of +50 kV. The photoelectrons are accelerated to weakly relativistic energies before being scattered at the foil. The atomic nuclei of the scattering target induce a magnetic field in the rest frame of the electrons, which introduces a spin-dependent term into the scattering cross section. This term depends only on the projection of the spin onto the direction of the magnetic field. As a result, the Mott polarimeter is sensitive only to transversal spin polarization. To convert an initially longitudinal spin polarization into a transverse one that can be detected, an electron-optical 90° bender is used. Two semiconductor detectors are placed symmetrically around the incident electron beam at angles of ±120° to register elastically backscattered electrons. If the photoelectrons are longitudinally unpolarized, the upper and lower detector register the same count rates, *I*_u_ and *I*_l_. An asymmetry in the count rates reflects a nonzero spin polarization. This asymmetry, *A* = (*I*_u_ − *I*_l_)/(Iu − Il), is related to the spin polarization *P = A/S* via the Sherman function S= 0.18, which quantifies the analyzing power of the polarimeter.

During the measurement, the light polarization is alternated between s-linear, cw circular, and ccw circular by rotation of a quarter-wave plate (QWP). At each QWP position, about 10^4^ electrons are collected. The measurement position alternates between the sample and a molybdenum reference mounted directly underneath the sample after each full rotation of the QWP. Since molybdenum emits unpolarized electrons regardless of light polarization, this allows for correction of instrumental asymmetries. To ensure proper alignment and stability of the polarimeter, the spin polarization of photoelectrons emitted from a sputter-cleaned Au (111) crystal surface is measured before and after each measurement. This surface emits photoelectrons with a well-defined spin polarization of about P=±25% under cw and ccw circularly polarized light. Only measurements that match these values before and after the sample measurement are considered valid. The measurements are conducted under ultrahigh vacuum conditions at a base pressure of less than 5 × 10^−9^ mbar. The entire setup is placed inside of three Helmholtz coils, which compensate for the earth’s magnetic field. A permalloy shielding reduces the residual magnetic fields to less than ∼20 μT.

The results of the spin polarization measurements are plotted as histograms, where each count in a specific spin polarization bin represents a single measurement, including about 10^4^ electrons. The histograms typically contain 200 to 300 individual measurements, i.e., about 10^6^ electrons in total and were acquired over an integration time of two hours. Gaussian curves are fitted to these distributions, and the average spin polarization and its uncertainty are extracted as the expectation value and the full width at half maximum.

## Supplementary Material

Appendix 01 (PDF)

## Data Availability

All study data are included in the article and/or *SI Appendix*.
