# Supplementary material for: Direct control of electron spin at an intrinsically chiral surface for highly efficient oxygen reduction reaction
Source: Proc Natl Acad Sci U S A. 2025 Feb 25;122(9):e2413609122. doi: 10.1073/pnas.2413609122 (PMC11892581; doi:10.1073/pnas.2413609122)
Supplement: Supplementary file 1 — Appendix 01 (PDF) [file pnas.2413609122.sapp.pdf]

Supporting Information for

Direct control of electron spin at an intrinsically chiral surface for highly efficient oxygen reduction reaction

Xia Wang[a,1,2], Mayra Peralta[a,1], Xiaodong Li[b,1], Paul V. Möllers[c], Dong Zhou[d], Patrick Merz[a], Ulrich Burkhardt[a], Horst Borrmann[a], Iñigo Robredo[a,e], Chandra Shekhar[a], Helmut Zacharias[c], Xinliang Feng[b,f,2], Claudia Felser[a,2]

[a]Department of Topological Quantum Chemistry, Max-Planck-Institute for Chemical Physics of Solids, 01187, Dresden, Germany;
[b]Centre for Advancing Electronics Dresden, Faculty of Chemistry and Food Chemistry, Technische Universität Dresden, 01062, Dresden, Germany;
[c]Center for Soft Nanoscience, University of Münster, 48149, Münster, Germany;
[d]Tsinghua Shenzhen International Graduate School, Tsinghua University, 518055, Shenzhen, China;
[e]Donostia International Physics Center, 20018, Donostia-San Sebastian, Spain;
[f]Department of Synthetic Materials and Functional Devices, Max Planck Institute of Microstructure Physics, 06120, Halle, Germany

[1]These authors contributed equally to this work.

[2]To whom correspondence may be addressed.

Email: xia.wang@cpfs.mpg.de; xinliang.feng@tu-dresden.de; Claudia.Felser@cpfs.mpg.de

This PDF file includes:

Supplementary Notes 1 to 2
Figures S1 to S20
References

Supplementary Note 1

We simulate the transmission through a finite central region of TH PdGa-A and TH PdGa-B with Hamiltonian $H_c^{A(B)}$, connected to two semi-infinite Ohmic contacts with Hamiltonian $H_{TL(BL)}$.
This setup is described by the equation(1):

$$H = H_0 + V = \begin{pmatrix} H_{TL} & 0 & 0 \\ 0 & H_c^{A(B)} & 0 \\ 0 & 0 & H_{BL} \end{pmatrix} + \begin{pmatrix} 0 & V_{TL-c} & 0 \\ V_{c-TL} & 0 & V_{BL-c} \\ 0 & V_{c-BL} & 0 \end{pmatrix}, \qquad (1)$$

Where H_0 is the Hamiltonian of the central region and the leads, and V contains the interactions between the TH PdGa central region and the contacts, with TL(BL) referring to the Top (Bottom) lead. The scattering problem is solved in the linear transport regime to calculate the spin-dependent transport through the central region that can be used to calculate the spin asymmetry as explained in the main text.

The Hamiltonian of the central region is constructed using a tight-binding model for space group NO.198.(2-4) The unit cell is chosen to have four sites, each carrying a single s-orbital. The positions of these sites are as follows: A at $e_{A(B)}(u,u,u)$, B at $e_{A(B)}(1/2+u, 1/2-u, -u)$, C at $e_{A(B)}(-u, 1/2+u, 1/2-u)$, and D at $e_{A(B)}(1/2-u, -u, 1/2+u)$, where $e_{A(B)}$ is 1 or -1 for TH PdGa-A and TH PdGa-B, respectively. DFT calculations indicate that the projected density of states (PDOS) and the differential charge distribution in TH PdGa are predominantly influenced by Pd d-orbitals (Supplementary Fig.15). Therefore, we focus only on Pd atoms in our calculations, with $u = 0.14$.

The Hamiltonian in reciprocal space(2-4) expressed in the basis $|A \uparrow\downarrow\rangle, |B \uparrow\downarrow\rangle, |C \uparrow\downarrow\rangle, |D \uparrow\downarrow\rangle$, is:

$$H_\phi = \begin{pmatrix} \varepsilon & f_{k_y,k_x} & g_{k_z,k_y} & h_{k_x,k_z} \\ f^*_{k_y,k_x} & \varepsilon & \tilde{h}_{k_x,k_z} & \tilde{g}_{k_z,k_y} \\ g^*_{k_z,k_y} & \tilde{h}^*_{k_x,k_z} & \varepsilon & \tilde{f}_{k_y,k_x} \\ h^*_{k_x,k_z} & \tilde{g}^*_{k_z,k_y} & \tilde{f}^*_{k_y,k_x} & \varepsilon \end{pmatrix} \qquad (2)$$

where the functions f_{k_i,k_j}, g_{k_i,k_j}, and h_{k_i,k_j} are defined as:

$$\begin{aligned} f_{k_i,k_j} &= 2e^{-ik_i/2}[tcos(\tfrac{k_j}{2})S_0 + i\lambda_z cos(\tfrac{k_j}{2})S_z + \lambda_x sin(\tfrac{k_j}{2})S_x + i\lambda_y cos(\tfrac{k_j}{2})S_y], \\ \tilde{f}_{k_i,k_j} &= 2e^{-ik_i/2}[tcos(\tfrac{k_j}{2})S_0 - i\lambda_z cos(\tfrac{k_j}{2})S_z + \lambda_x sin(\tfrac{k_j}{2})S_x + i\lambda_y cos(\tfrac{k_j}{2})S_y], \\ g_{k_i,k_j} &= 2e^{-ik_i/2}[tcos(\tfrac{k_j}{2})S_0 + i\lambda_y cos(\tfrac{k_j}{2})S_z + i\lambda_z cos(\tfrac{k_j}{2})S_x + \lambda_x sin(\tfrac{k_j}{2})S_y], \\ \tilde{g}_{k_i,k_j} &= 2e^{ik_i/2}[tcos(\tfrac{k_j}{2})S_0 - i\lambda_y cos(\tfrac{k_j}{2})S_z + i\lambda_z cos(\tfrac{k_j}{2})S_x + \lambda_x sin(\tfrac{k_j}{2})S_y], \\ h_{k_i,k_j} &= 2e^{-ik_i/2}[tcos(\tfrac{k_j}{2})S_0 + \lambda_x sin(\tfrac{k_j}{2})S_z + i\lambda_y cos(\tfrac{k_j}{2})S_x + i\lambda_z cos(\tfrac{k_j}{2})S_y], \\ \tilde{h}_{k_i,k_j} &= 2e^{-ik_i/2}[tcos(\tfrac{k_j}{2})S_0 + \lambda_x sin(\tfrac{k_j}{2})S_z + i\lambda_y cos(\tfrac{k_j}{2})S_x - i\lambda_z cos(\tfrac{k_j}{2})S_y], \end{aligned} \qquad (3)$$

Here, S_x, S_y, and S_z are the spin Pauli matrices, S_0 is the identity matrix, t is the hopping parameter, and ε is the onsite energy. The intrinsic spin-orbit coupling (SOC) is incorporated using parameters $\lambda_x, \lambda_y, \lambda_z$. A second-nearest-neighbor term representing hopping between adjacent unit cells is added to the Hamiltonian to reproduce the energy offset between the fourfold fermion at the Γ point and the sixfold degenerate double spin -1 at the R point:

$t_2[cos(k_x) + cos(k_y) + cos(k_z)]1_{8\times8}$

The contacts are modeled as semi-infinite Ohmic leads made of the same material as the central region, but without SOC, meaning $\lambda_x = \lambda_y = \lambda_z = 0$.

The calculations were performed with an onsite energy $\varepsilon = 0$ eV, a nearest-neighbor (NN) hopping $t = 0.6$ eV, and next-nearest-neighbor (NNN) hopping $t_2 = 0.13$. To simulate an imperfect coupling between the central region and the contacts, a small was introduced by setting the onsite energy of the contacts as $\varepsilon_c = 0.25$ eV. The intrinsic SOC parameters are $\lambda_x = \lambda_y = 0.02$eV and $\lambda_z = 0.04$ eV. These values of the parameters were previously fitted to the bands.(5, 6)

The spin polarization was quantified as the asymmetry between the up and down spins transmitted through the crystal, expressed as:

$$\mathcal{P} = \frac{T_{\uparrow\uparrow}+T_{\downarrow\uparrow}-T_{\uparrow\downarrow}-T_{\downarrow\downarrow}}{T_{\uparrow\uparrow}+T_{\uparrow\downarrow}+T_{\downarrow\uparrow}+T_{\downarrow\downarrow}} \times 100 \quad (4)$$

where the sub-indices with arrows represent the spin directions of the incoming and outgoing electrons transported through the central region.

To assess the relative importance of chirality and intrinsic SOC in spin polarization, we performed calculations for a non-chiral material with the same central region geometry (four sites in the unit cell). Specifically, the sites were located at: A $(0,0,0)$, B $(1/2,1/2,0)$, C $(0,1/2,1/2)$, and D $(1/2,0,1/2)$. As in the previous case, the contacts are semi-infinite Ohmic leads made of the same material as the central region, but without SOC.

During the oxygen reduction reaction (ORR), electrons are transferred from the electrode to oxygen intermediates adsorbed on the electrode surface(7), which are influenced by the applied potential. These electron transfer processes also depend on the Fermi level of both the electrode and the oxygen species. Therefore, the electronic and transport properties of the catalyst at the Fermi level play a pivotal role in ORR performance.(8-10) The calculations were carried out using the Landauer formalism(1), with a simplified model that includes only the Pd sites, which dominate the electronic and transport properties of the electrode near the Fermi level (*SI Appendix*, Fig. S15). Specifically, we examine spin-dependent electron transfer (ET), considering a SOC gap of approximately 40 meV between the 4-fold and the 2-fold fermions at the G point. Our results indicate a detectable spin-dependent electron transfer through the Pd sites in the TH PdGa crystal. This observation serves as a qualitative indicator of spin-dependent ET from Pd sites to the adsorbed species during ORR(11, 12).

Supplementary Note 2

The thermodynamic calculation is based on DFT as implemented in the Vienna Ab initio Simulation Package (VASP).(13) The exchange-correlation potential was described by the generalized gradient approximation (GGA) within the framework of Perdew-Burke-Ernzerhof (PBE) functional.(14) The DFT-D3 method was employed to calculate the van der Waals (vdW) interaction.(15) The parameters of dipole correction were applied for the calculation of slab models. Electronic energies were computed with the tolerance of 1×10^{-5} eV and total force of 0.01 eV/Å. Monkhorst−Pack k-meshs of 3 × 4 × 1 k-points and 5 × 5 × 1 k-points were used for TH PdGa slab and Pt slab in the structural relaxation, respectively. A kinetic cutoff energy of 450 eV was adopted to obtain the precise energy. The Pt (111) slabs were modeled by the corresponding exposed surface along (111) direction with the thickness of 7 atomic layers (the bottom three layers were fixed).(16) TH PdGa (111) slab model with four-unit-cell thickness was constructed, in which the bottom two unit cells were immobilized to simulate the bulk. For the TH PdGa (111) slab, various exposed surfaces were constructed to obtain the most stable configuration. A vacuum space of 15 Å was inserted in z direction to avoid interactions between periodic images.

The computational hydrogen electrode (CHE)(17) model was used to calculate the Gibbs free energy change (ΔG) of O_2 reduction reaction steps:

$G = E_{DFT} + E_{ZPE} – TS$
$E_{ZPE} = \sum_i 1/2\ h\nu_i$
$\Theta_i = h\nu_i / k$
$S = \sum_i R[\ln (1-e^{-\Theta i/T})^{-1} + \Theta_i/T\ (e^{\Theta i/T} - 1)^{-1}]$

where E_{DFT} is the electronic energy calculated for specified geometrical structures, E_{ZPE} is the zero-point energy, S is the entropy, h is the Planck constant, v is the computed vibrational frequencies, Θ is the characteristic temperature of vibration, k is the Boltzmann constant, and R is the molar gas constant. For adsorbates, all 3N degrees of freedom were treated as frustrated harmonic vibrations with negligible contributions from the catalysts' surfaces. Thus, free energy changes relative to an initial state can be represented by:

$\Delta G\ [OOH^*] = G\ [OOH^*] + 3 \times G\ [H+ + e-] – (G\ [^*] + G\ [O_2] + 4 \times G\ [H^+ + e^-])$
$\Delta G\ [O^*] = G\ [O^*] + G\ [H_2O] + 2 \times G\ [H+ + e-] – (G\ [^*] + G\ [O_2] + 4 \times G\ [H^+ + e^-])$
$\Delta G\ [OH^*] = G\ [OH^*] + G\ [H_2O] + G\ [H+ + e-] – (G\ [^*] + G\ [O_2] + 4 \times G\ [H^+ + e^-])$
$G\ [H+ + e-] = 1/2\ G[H_2] – eU$

where * is the substrate, U is the applied overpotential and *e* is the elementary charge. In this study, U = 1.23 V versus reversible hydrogen electrode was performed for ORR. The free energy of the $(H^+ + e^-)$ pair is related to that of 1/2 H_2 in the gas-phase and that for O_2 is expressed as $G[O_2]=4.92$ eV + $2G[H_2O]-2G[H_2]$ to avoid DFT calculations of the energy of O_2, for which the electronic structure is not well described by DFT methods.(18)

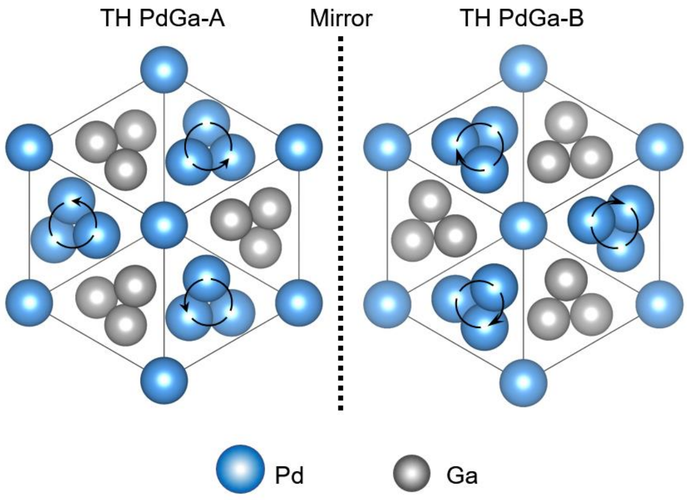

Fig. S1. Schematic illustrating the surface chirality of both TH PdGa-A and TH PdGa-B enantiomers on the (111) crystal surface.

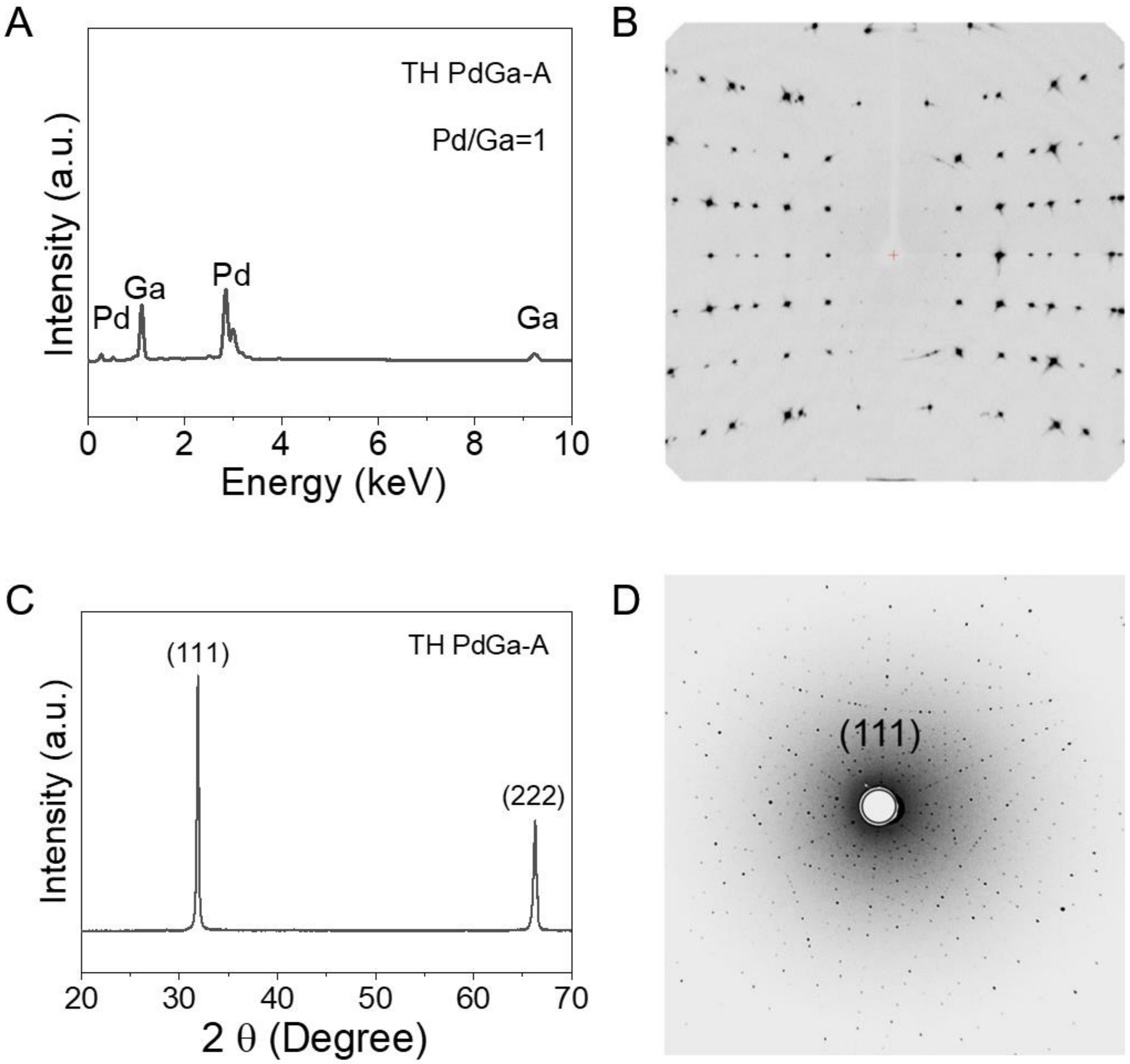

Fig. S2. *(A)* EDS spectrum of TH PdGa-A. *(B)* Single-crystal XRD pattern of TH PdGa-A along c axis. The high quality of the TH PdGa-A crystal is confirmed by the sharp and clear diffraction spots. *(C)* XRD pattern of plate-like TH PdGa-A single crystal. *(D)* Laue X-ray diffraction pattern of TH PdGa-B crystal. The Laue X-ray diffraction displays a single (111) plane pattern, confirming the good single crystallinity of TH PdGa-B without twinning or domains.

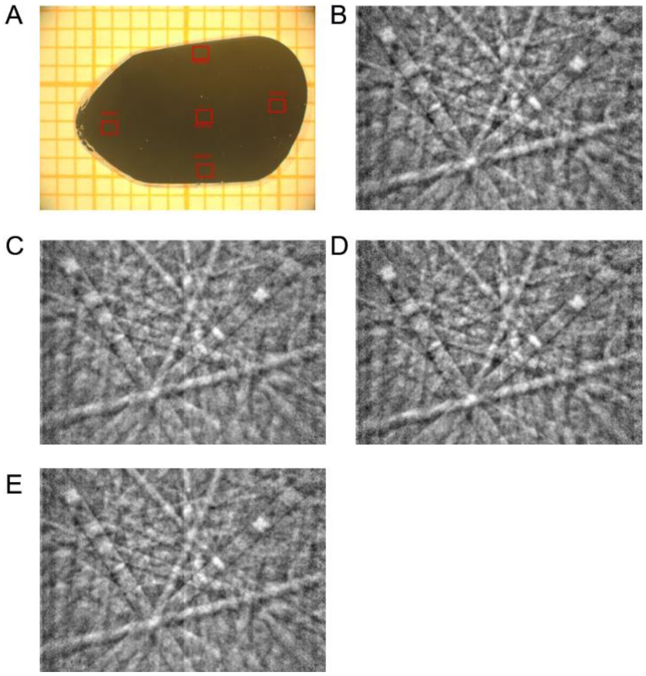

Fig. S3. *(A)* Optical image of TH PdGa-A. EBSD patterns detected at site *(B)* EBSD02, *(C)* EBSD03, *(D)* EBSD04 and *(E)* EBSD05. The red rectangles in panel (A) indicate the detection sites for EBSD.

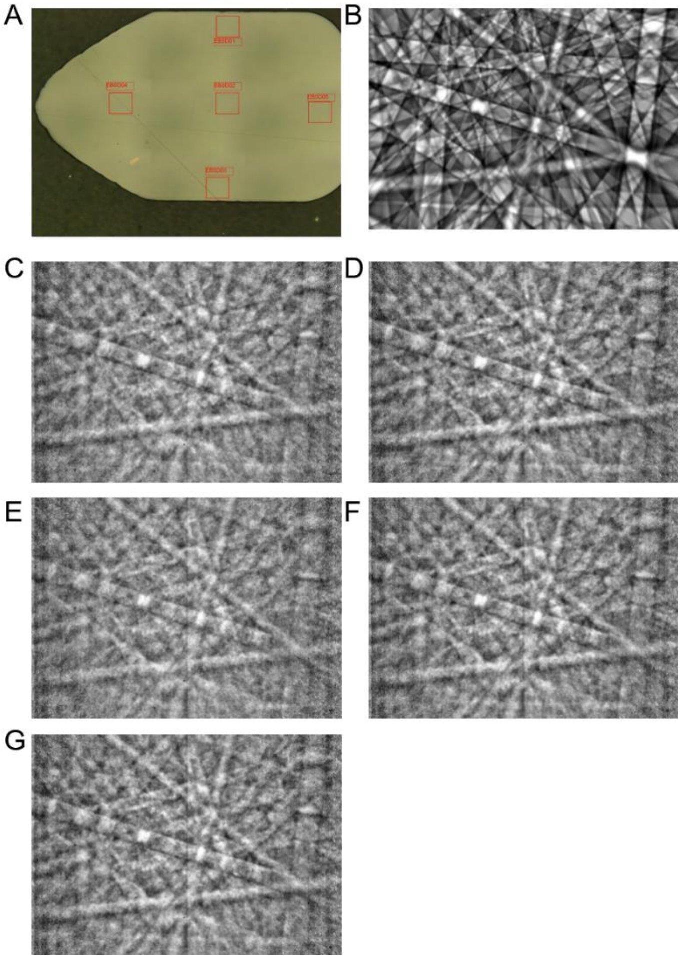

Fig. S4. *(A)* Optical image of TH PdGa-B. *(B)* Simulated EBSD pattern of TH PdGa-B. EBSD patterns detected at sites *(C)* EBSD01, *(D)* EBSD02, *(E)* EBSD03, *(F)* EBSD04 and *(G)* EBSD05. The red rectangles in panel (A) indicate the detection sites for EBSD. *Note:* The assignment of the handedness is based on the quantitative comparison of experimental and calculated patterns of TH PdGa-A and TH PdGa-B. The handness of TH PdGa is defined by the coordinates of Pd and Ga in the Wyckoff site 4(*a*) *xxx*. For TH PdGa-A, the coordinates are x(Pd) = 0.1422 and x(Ga) ≈ 0.8427,(19, 20) while for TH PdGa-B, x(Pd) = 1- 0.1422 = 0.8578 and x(Ga) = 1 - 0.8427 = 0.1573. The simulation of the EBSD pattern is realized within the Bloch wave approach, where the consideration of dynamical electron scattering leads to chirality dependent contributions(21, 22). The cross-correlation coefficients, r_{+E} and r_{-E} ($0 \leq r_{+E}, r_{-E} \leq 1$), measure the match between experimental and calculated EBSD patterns for TH PdGa-A and TH PdGa-B. The difference, $\Delta r = r_{+E} - r_{-E}$, with $|\Delta r| \geq 0.01$, is significant and allows assignment of the handedness based on the EBSD pattern if the match between experimental and simulated pattern results in $r_m = (r_{+E} + r_{-E})/2 > 0.5$. This method has been validated by consistent handedness determination via single crystal XRD and EBSD in recent publications(23, 24). The evaluation of the EBSD patterns give typical values of $r_m \approx 0.58$ for both TH PdGa-A and TH PdGa-B (Fig. S3-S4). The EBSD results for TH PdGa-A show $\Delta r > +0.006$ (Fig. S3), and for TH PdGa-B, $\Delta r < -0.011$ (Fig. S4). It is noteworthy that the evaluation of EBSD03 (Fig. S4) yields $\Delta r = -\ 0.001$, which still indicates TH PdGa-B in this region. The assignment of TH PdGa-A with $\Delta r = +0.012$ is confirmed by the crystal structure solution from single crystal XRD data.

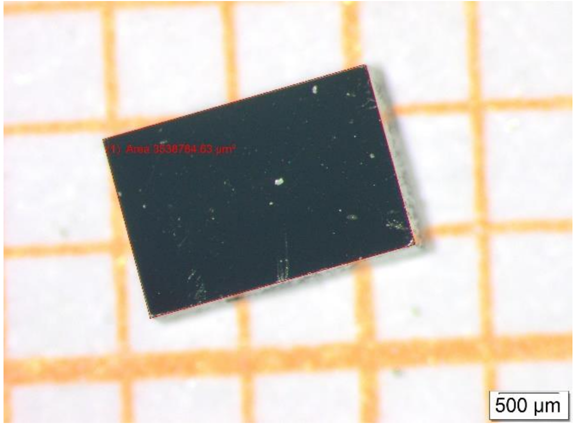

Fig. S5. Optical image of the TH PdGa-A working electrode fabricated directly from a single crystal. The effective surface area, measured directly, is indicated on the image.

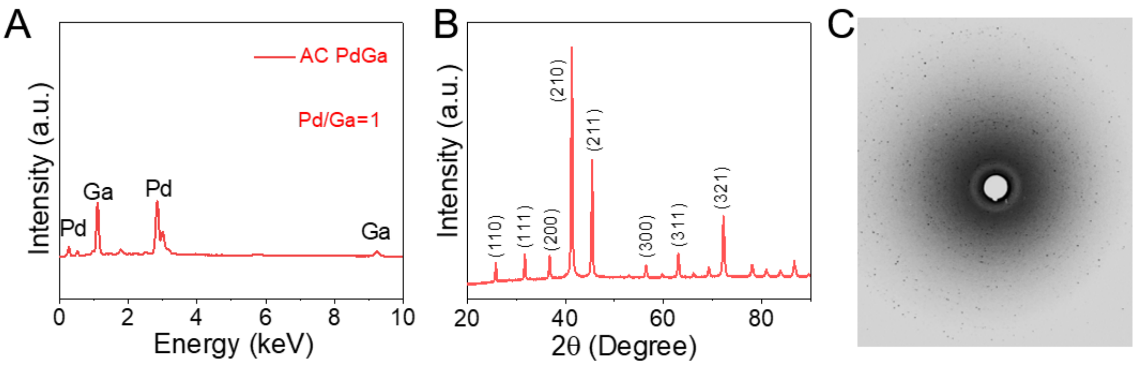

Fig. S6. *(A)* EDS of AC PdGa. *(B)* X-ray diffraction pattern of AC PdGa. *(C)* Laue X-ray diffraction pattern of AC PdGa.

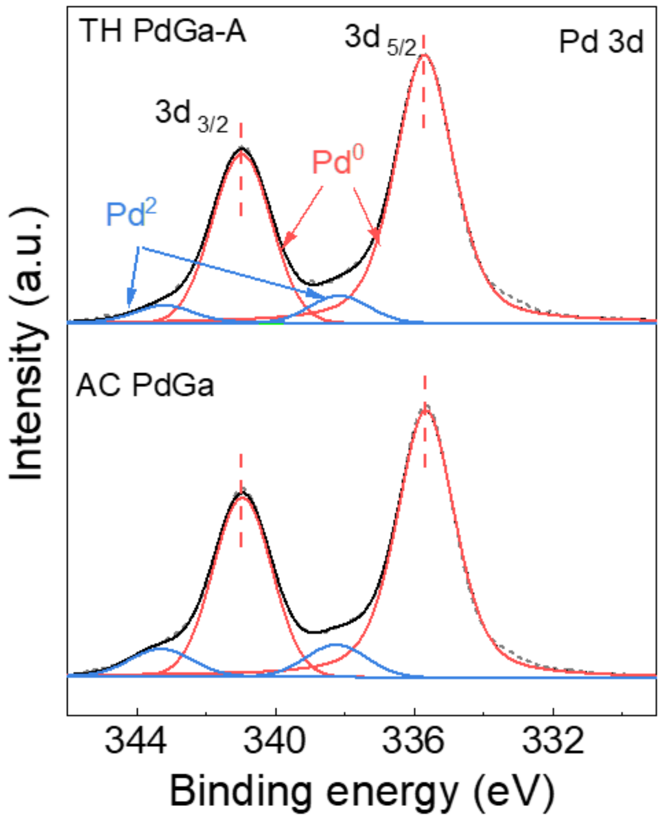

Fig. S7. High-resolution XPS of Pd 3d for TH PdGa-A and AC PdGa. The high-resolution Pd 3d XPS show similar valence states of Pd for both TH PdGa-A and AC PdGa, with the majority of Pd existing in a metallic state.

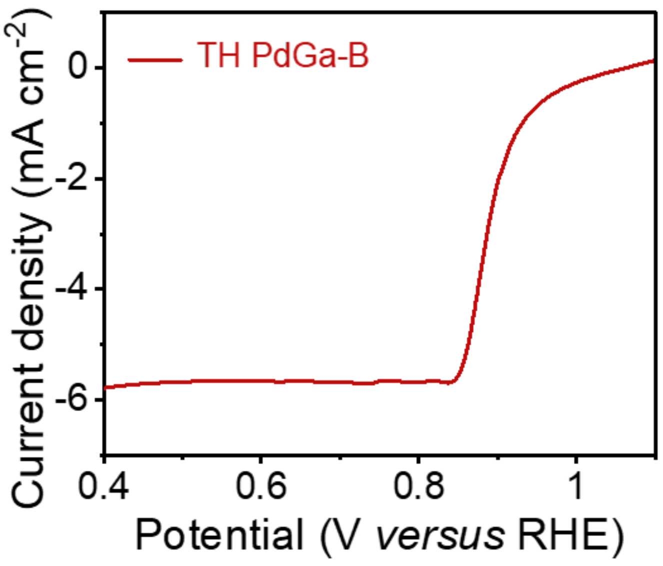

Fig. S8. LSV curve of TH PdGa-B. The LSV curve shows that TH PdGa-B exhibits similar ORR activity to TH PdGa-A. Therefore, only the ORR performance of TH PdGa-A was measured and used to represent both enantiomers in the subsequent tests.

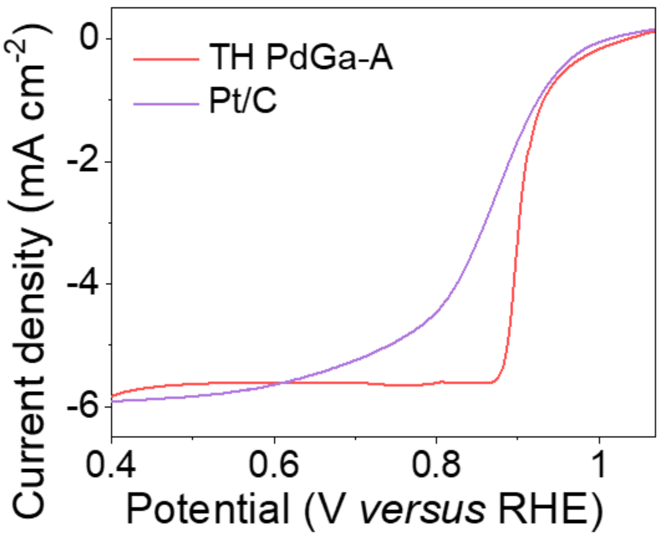

Fig. S9. LSV curves of TH PdGa-A and Pt/C. The benchmark Pt/C demonstrated an E_{onset} of 0.99 V *versus* RHE and an $E_{1/2}$ of 0.86 V *versus* RHE. The J_k of Pt/C at 0.85 V *versus* RHE was 7.5 mA cm^{-2}. *Note:* Pt/C is in powder form, and the Pt/C electrode is prepared by the drop-cast method on the rotating disk electrode. In contrast, a bulk crystal of TH PdGa-A is directly used as the electrode. Therefore, the electron behavior during interfacial and bulk transport differs for the two types of electrodes. In addition, the Pt/C thin film electrode has a larger electrochemical surface area compared to the bulk electrode with a smooth surface, indicating that TH PdGa outperforms Pt/C despite its lower electrochemical surface area.

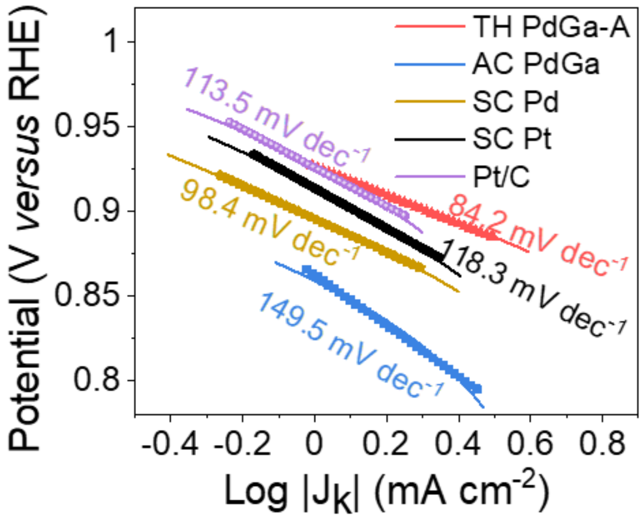

Fig. S10. Tafel plots of TH PdGa-A, AC PdGa, SC Pd, and SC Pt, and Pt/C.

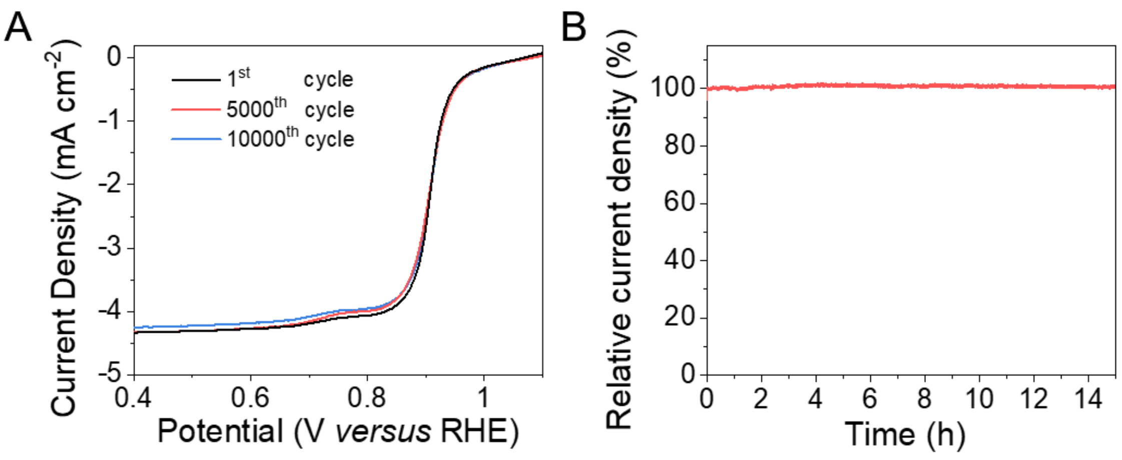

Fig. S11. *(A)* LSV curves of TH PdGa-A before, after 5000 cycles, and after 10000 cycles at a rotation rate of 900 rpm. *(B)* Chronoamperometric response of TH PdGa-A at 0.5 V *versus* RHE.

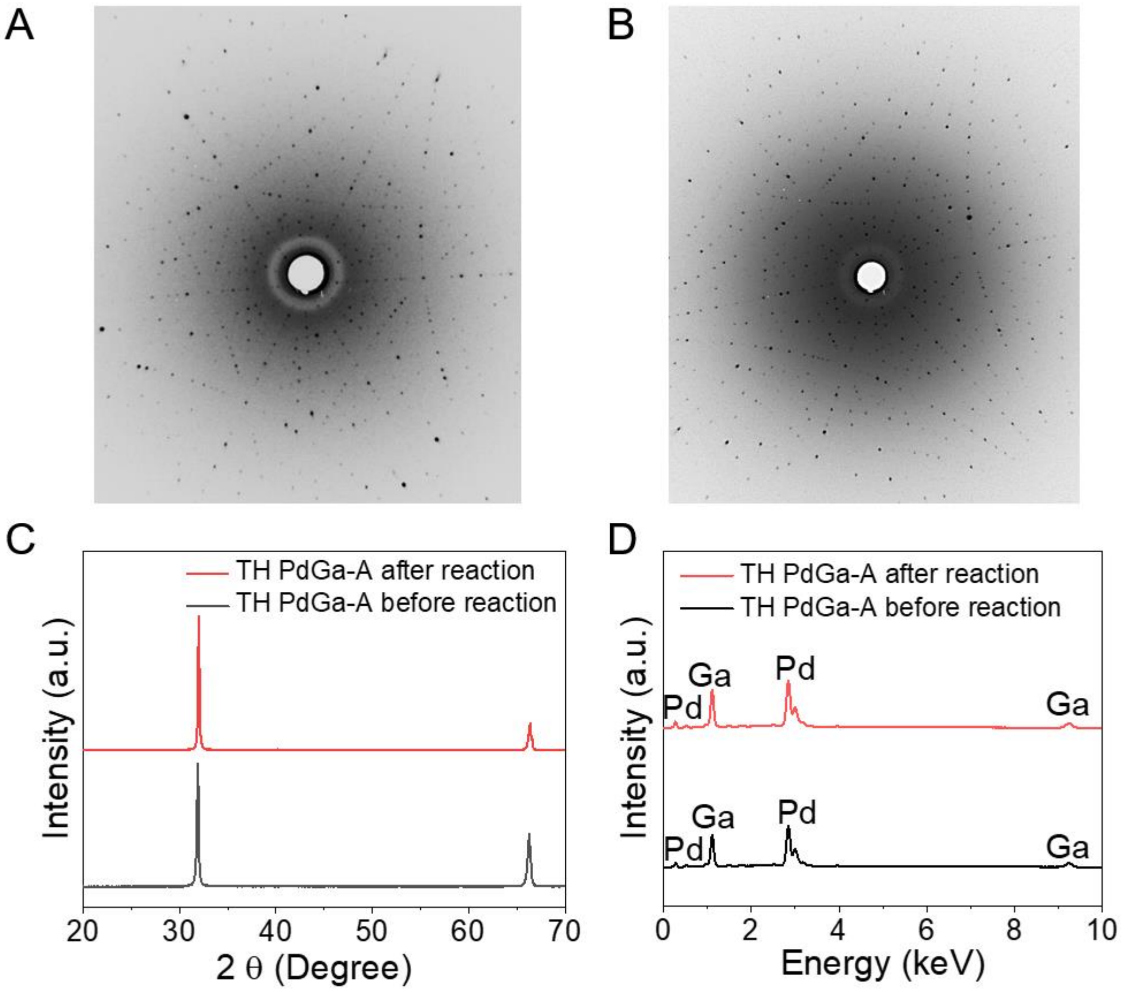

Fig. S12. Laue X-ray diffraction of TH PdGa-A *(A)* before cycling, and *(B)* after cycling. *(C)* XRD pattern of TH PdGa-A crystal before reaction (black) and after reaction (red). *(D)* EDS of TH PdGa-A before reaction (black) and after reaction (red). The Pd/Ga ratio are 1 for TH PdGa-A before reaction (black) and after reaction (red).

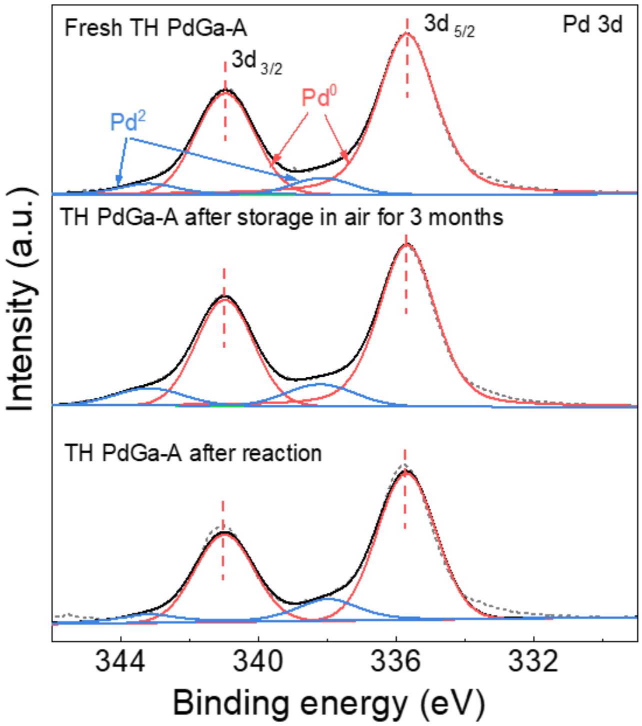

Fig. S13. High-resolution XPS of Pd 3d.

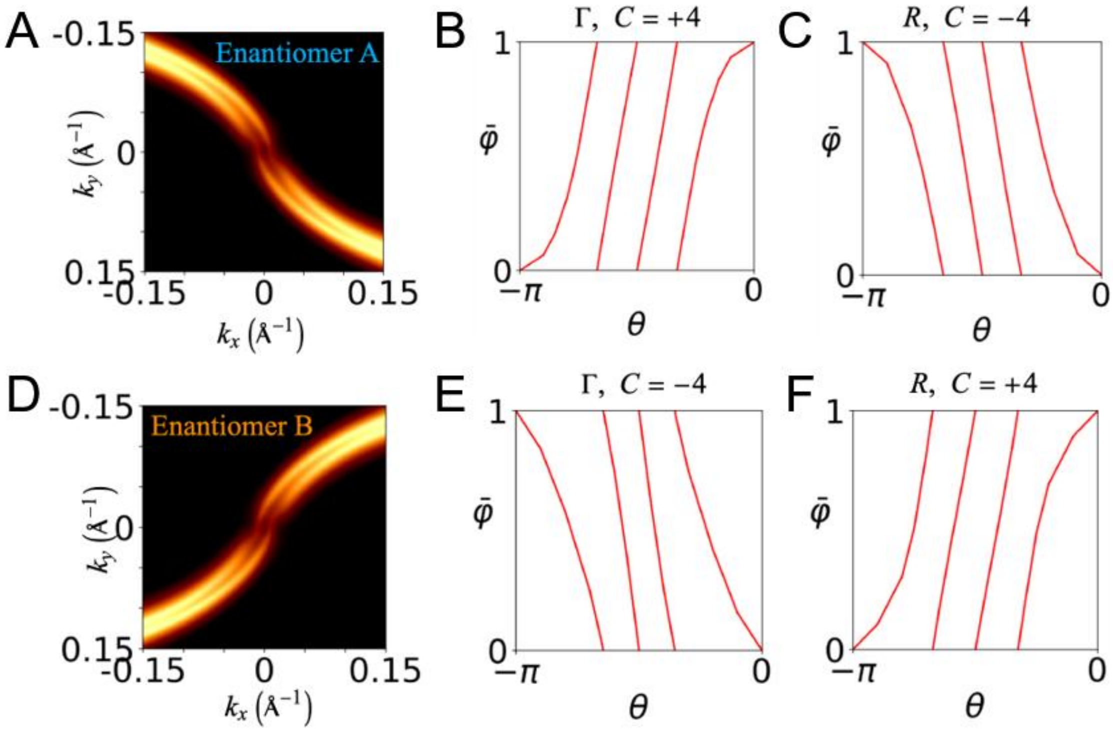

Fig. S14. Fermi arc TSS connecting G and R points for *(A)* TH PdGa-A, and *(D)* TH PdGa-B. Wilson loops for spheres centered on G with $\boldsymbol{N_{occ}} = \mathbf{4}$ for *(B)* TH PdGa-A, and *(E)* TH PdGa-B. Wilson loops for spheres centered on R with $\boldsymbol{N_{occ}} = \mathbf{2}$ for *(C)* TH PdGa-A, and *(F)* TH PdGa-B. The Chern number indicates that G point is a source (sink) and R point is a sink (source) of Berry curvature.

The Chern number is calculated as:

$$\boldsymbol{C} = \sum_{\boldsymbol{m}=\mathbf{1}}^{\boldsymbol{N_{occ}}} \int_{\mathcal{M}} \boldsymbol{dS} \cdot \boldsymbol{F_m}(\boldsymbol{k})$$

where the sum is over all the occupied bands with $\boldsymbol{m} = \mathbf{1}, \mathbf{2}, \ldots, \boldsymbol{N_{occ}}$, and $\boldsymbol{N_{occ}}$ is the number of occupied bands. $\boldsymbol{F_m}(\boldsymbol{k}) = \boldsymbol{\nabla_k} \times \boldsymbol{A_m}(\boldsymbol{k})$ is the Berry curvature, and $\boldsymbol{A_m}(\boldsymbol{k}) = -\boldsymbol{i}\langle \boldsymbol{u_m}(\boldsymbol{k}) | \boldsymbol{\nabla_k} | \boldsymbol{u_m}(\boldsymbol{k}) \rangle$ is the Berry connection. The integration is performed over a sphere that encloses the manifold $\mathcal{M}$ using the Wilson loop.

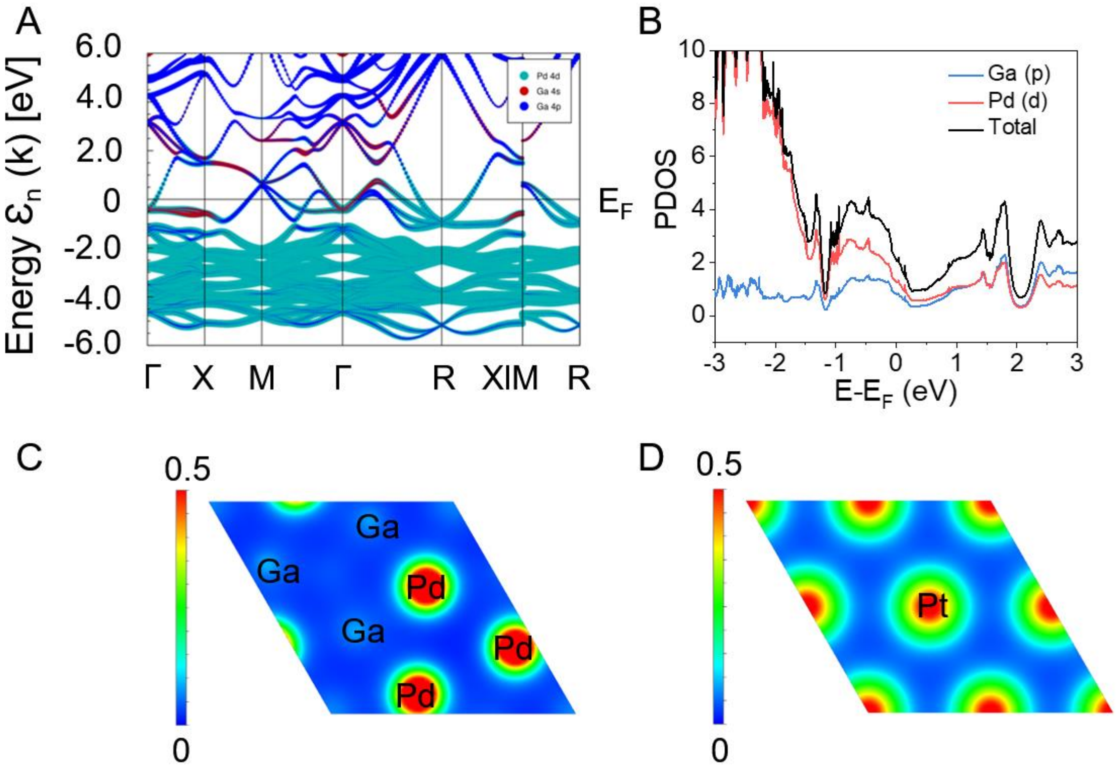

Fig. S15. *(A)* Orbital weights of TH PdGa. *(B)* PDOS plots for TH PdGa. *(C)* Differential charge distribution for TH PdGa. *(D)* Differential charge distribution for Pt. The orbital weight of Pd is higher than that of Ga, being twice as large near the Fermi level and much higher below it (Panels A-B). Additionally, the electron density distribution on the TH PdGa crystal slab shows electron accumulation around the Pd atoms, likely due to charge transfer between Ga and Pd (Panels C-D).

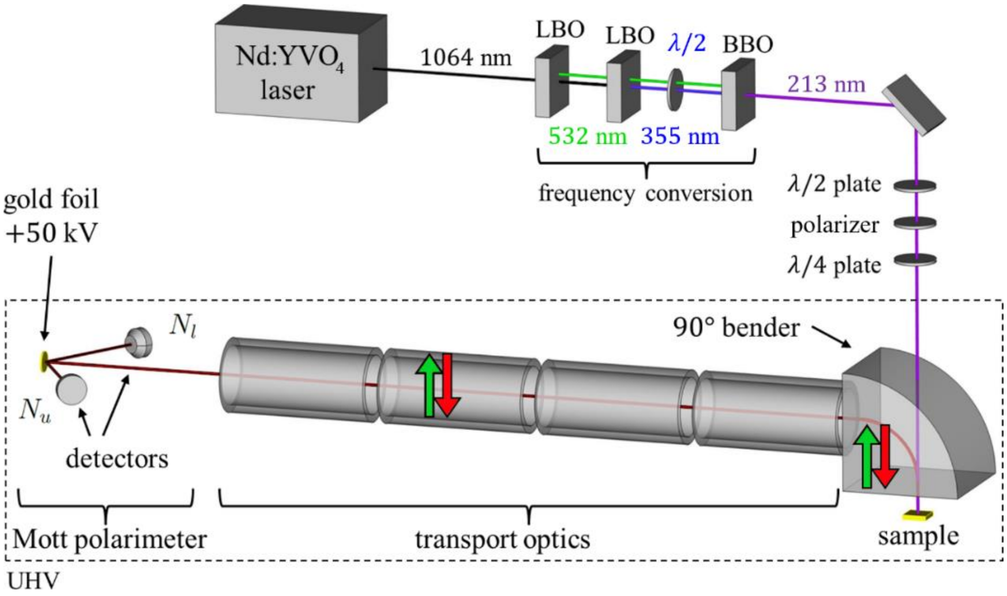

Fig. S16. Scheme of the Mott scattering setup. Reprinted with permission.(25)

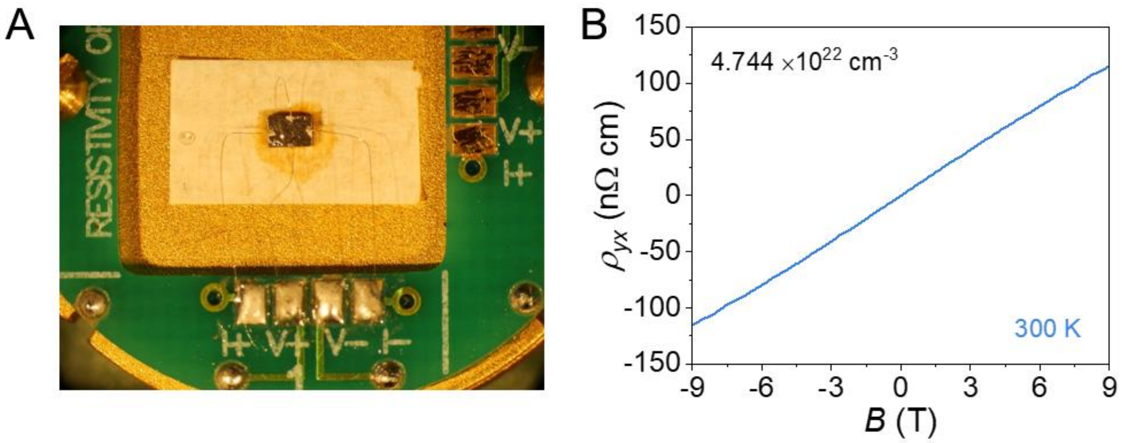

Fig. S17. *(A)* Configuration of the Hall resistivity measurement for TH PdGa. *(B)* Magnetic-field dependence of the Hall resistivity for TH PdGa at 300 K. The carrier concentration (*n*) is calculated using $n\ (T) = 1/[e^{*}R_{H}(T)]$, where e is the electronic charge and R_H is the Hall coefficient.

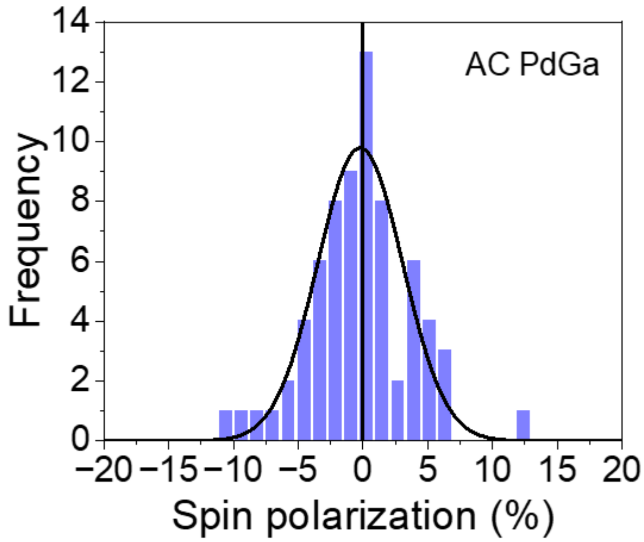

Fig. S18. Photoelectron spin polarization distributions measured on AC PdGa.

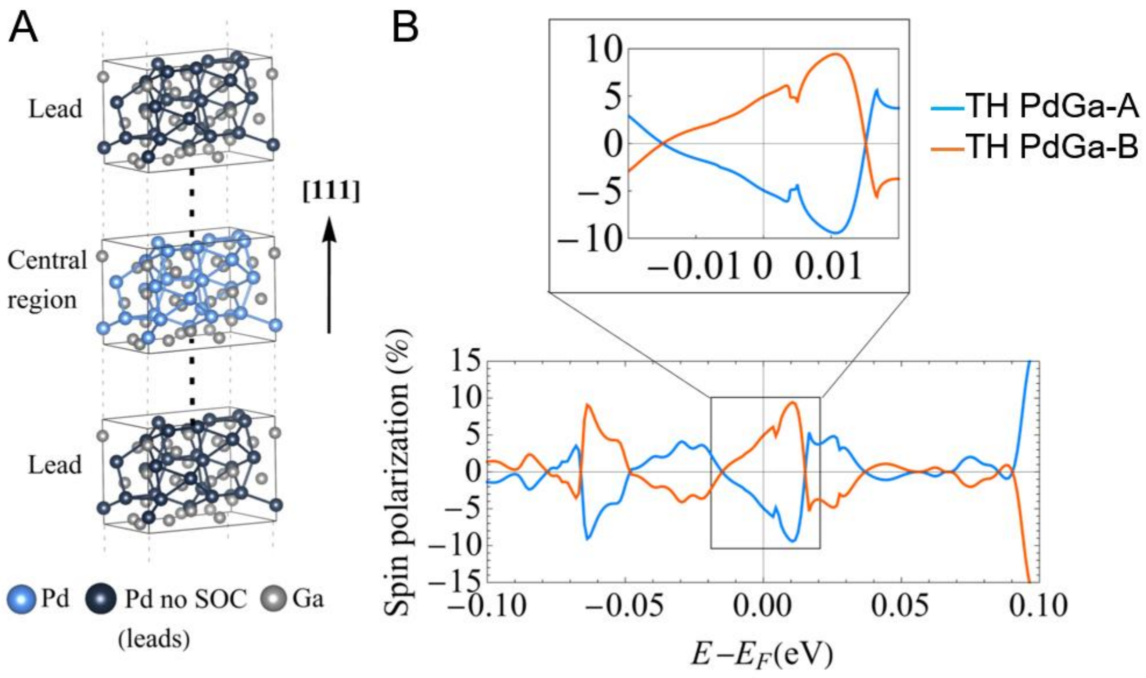

Fig. S19. *(A)* Depiction of the system used for theoretical calculation, representing the central region and the leads in the [111] direction. *(B)* The corresponding spin polarization calculated for TH PdGa. In all plots, the parameters used are: onsite energy ε = 0 eV, hopping t = 0.6 eV, and intrinsic SOC parameters $\boldsymbol{\lambda_x = \lambda_y = 0.02}$ eV and $\boldsymbol{\lambda_z = 0.04}$ eV. For the contacts, the onsite energy is $\boldsymbol{\varepsilon_c = 0.25}$ eV and the hopping parameter is t = 0.6 eV.

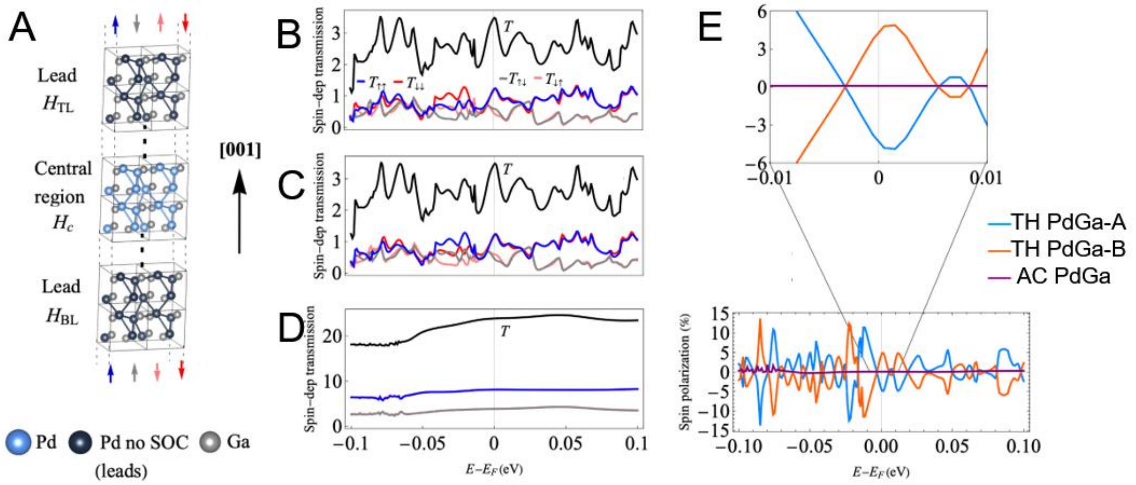

Fig. S20. *(A)* Depiction of the system used for theoretical calculation, representing the central region and the leads in the [001] direction. Arrows at the top and the bottom represent the spin dependent transmission. *(B-D)* Spin dependent transmission for TH PdGa-A, TH PdGa-B, and AC PdGa, respectively. *(E)* The corresponding spin polarization calculated for TH PdGa-A, TH PdGa-B, and AC PdGa. In all plots, the parameters used are: onsite energy ε = 0 eV, hopping t = 0.6 eV, and intrinsic SOC parameters $\boldsymbol{\lambda_x = \lambda_y = 0.02}$ eV and $\boldsymbol{\lambda_z = 0.04}$ eV. For the contacts, the onsite energy is $\boldsymbol{\varepsilon_c = 0.25}$ eV and the hopping parameter is t = 0.6 eV. Notably, the theoretical modeling excludes decoherence or thermal fluctuations, which may influence the polarizations observed in experiments conducted at room temperature.

References

1. C. W. Groth, M. Wimmer, A. R. Akhmerov, X. Waintal, Kwant: a software package for quantum transport. *New J. Phys.* **16**, 063065 (2014).
2. G. Chang *et al.*, Unconventional chiral fermions and large topological Fermi arcs in RhSi. *Phys. Rev. Lett.* **119**, 206401 (2017).
3. Y. Sun, Q. Xu, Y. Zhang, C. Le, C. Felser, Optical method to detect the relationship between chirality of reciprocal space chiral multifold fermions and real space chiral crystals. *Phys. Rev. B* **102**, 104111 (2020).
4. I. Robredo *et al.*, Cubic Hall viscosity in three-dimensional topological semimetals. *Phys. Rev. Res.* **3**, L032068 (2021).
5. P. Sessi *et al.*, Handedness-dependent quasiparticle interference in the two enantiomers of the topological chiral semimetal PdGa. *Nat. Commun.* **11**, 3507 (2020).
6. Q. Yang *et al.*, Monopole-like orbital-momentum locking and the induced orbital transport in topological chiral semimetals. *Proc. Natl. Acad. Sci. U.S.A.* **120**, e2305541120 (2023).
7. H. Reiss, The Fermi level and the redox potential. *J. Phys. Chem.* **89**, 3783-3791 (1985).
8. K. Kondou *et al.*, Fermi-level-dependent charge-to-spin current conversion by Dirac surface states of topological insulators. *Nat. Phys.* **12**, 1027-1031 (2016).
9. J. Nayak *et al.*, Electronic properties of topological insulator candidate CaAgAs. *J. Phys.: Condens. Matter* **30**, 045501 (2018).
10. J. Hu, S.-Y. Xu, N. Ni, Z. Mao, Transport of topological semimetals. *Annu. Rev. Mater. Res.* **49**, 207-252 (2019).
11. Y.-L. Lee, J. Kleis, J. Rossmeisl, Y. Shao-Horn, D. Morgan, Prediction of solid oxide fuel cell cathode activity with first-principles descriptors. *Energy Environ. Sci.* **4**, 3966-3970 (2011).
12. K. A. Stoerzinger *et al.*, Decreasing the Hydroxylation Affinity of La1–x Sr x MnO3 Perovskites To Promote Oxygen Reduction Electrocatalysis. *Chem. Mater.* **29**, 9990-9997 (2017).
13. G. Kresse, J. Furthmüller, Efficiency of ab-initio total energy calculations for metals and semiconductors using a plane-wave basis set. *Comput. Mater. Sci.* **6**, 15-50 (1996).
14. J. P. Perdew, K. Burke, M. Ernzerhof, Generalized gradient approximation made simple. *Phys. Rev. Lett.* **77**, 3865 (1996).
15. S. Grimme, J. Antony, S. Ehrlich, H. Krieg, A consistent and accurate ab initio parametrization of density functional dispersion correction (DFT-D) for the 94 elements H-Pu. *J. Chem. Phys.* **132**, 154104 (2010).
16. L. Gao *et al.*, Unconventional p–d hybridization interaction in PtGa ultrathin nanowires boosts oxygen reduction electrocatalysis. *J. Am. Chem. Soc.* **141**, 18083-18090 (2019).
17. A. A. Peterson, F. Abild-Pedersen, F. Studt, J. Rossmeisl, J. K. Nørskov, How copper catalyzes the electroreduction of carbon dioxide into hydrocarbon fuels. *Energy Environ. Sci.* **3**, 1311-1315 (2010).
18. J. K. Nørskov *et al.*, Origin of the overpotential for oxygen reduction at a fuel-cell cathode. *J. Phys. Chem. B* **108**, 17886-17892 (2004).
19. J. C. H. Spence, J. M. Zuo, M. O'Keeffe, K. Marthinsen, R. Hoier, On the minimum number of beams needed to distinguish enantiomorphs in X-ray and electron diffraction. *Acta Crystallogr. A* **50**, 647-650 (1994).
20. M. Armbrüster *et al.*, Refinement of the crystal structure of palladium gallium (1:1), PdGa. *Z. fur Krist. - New Cryst. Struct.* **225**, 617-618 (2010).
21. A. Winkelmann, C. Trager-Cowan, F. Sweeney, A. P. Day, P. Parbrook, Many-beam dynamical simulation of electron backscatter diffraction patterns. *Ultramicroscopy* **107**, 414-421 (2007).
22. A. Winkelmann *et al.*, Kikuchi pattern simulations of backscattered and transmitted electrons. *J Microsc* **284**, 157-184 (2021).
23. A. Winkelmann, G. Nolze, Point-group sensitive orientation mapping of non-centrosymmetric crystals. *Appl. Phys. Lett.* **106** (2015).
24. U. Burkhardt *et al.*, Absolute Structure from Scanning Electron Microscopy. *Sci. Rep.* **10**, 4065 (2020).

25. K. Ghosh *et al.*, Controlling chemical selectivity in electrocatalysis with chiral CuO-coated electrodes. *J. Phys. Chem. C* **123**, 3024-3031 (2019).
